# Nutrition label experience, obesity, high blood pressure, and high blood lipids in a cohort of 42,750 Thai adults

**DOI:** 10.1371/journal.pone.0189574

**Published:** 2017-12-13

**Authors:** Wimalin Rimpeekool, Vasoontara Yiengprugsawan, Martyn Kirk, Cathy Banwell, Sam-ang Seubsman, Adrian Sleigh

**Affiliations:** 1 National Centre for Epidemiology & Population Health, Research School of Population Health, Australian National University, Canberra, Australia; 2 School of Human Ecology, Sukhothai Thammathirat Open University, Nonthaburi, Thailand; 3 Centre for Research on Ageing, Health, and Wellbeing, Research School of Population Health, Australian National University, Canberra, Australia; 4 Australian Research Council–Centre for Research on Population Ageing Research (CEPAR), Canberra, Australia; University of Kentucky, UNITED STATES

## Abstract

**Introduction:**

Nutrition labels have been promoted for nearly two decades in Thailand to educate people about healthy eating and to combat nutrient-related non-communicable diseases (NCDs). But little is known about how nutrition labels are experienced and whether they are linked with better health. Our objective was to investigate the associations between nutrition label experience, obesity and nutrient-related NCDs in Thai consumers.

**Methods:**

A cross-sectional study was undertaken with a nationwide cohort of 42,750 distance learning Thai adult students enrolled in an Open University in 2013. We measured exposure as nutrition label experience (read, understand, use). Health outcomes were high blood pressure, high blood lipids, and high Body Mass Index (overweight at risk and obesity). Multivariate logistic regression was used to determine the association between nutrition label experience and health outcome adjusting for sociodemographic attributes, physical activity, smoking, and alcohol intake.

**Results:**

Frequent nutrition label use varied by cohort attributes and health outcomes and was least for those with low physical activity and high blood pressure. Being male, older, an urban resident or with low physical activity was associated with increasing high blood pressure and high blood lipids. Compared to those who read, understand and use nutrition labels, participants who did not (read, understand, and use), were more likely to report high blood pressure (Adjusted Odds Ratio 1.33; 1.17–1.51), high blood lipids (AOR 1.26; 1.14–1.39), and obesity (AOR 1.23; 1.13–1.33), but were not more likely to be overweight at risk (AOR 1.06; 0.97–1.16).

**Conclusions:**

We found cross-sectional associations between low nutrition label experience and increased likelihood of high blood pressure, high blood lipids, and obesity among Thai adults. Nutrition label education should be promoted as part of a public health approach to appropriate food choices and better lifestyles to reduce obesity and nutrient-related NCDs.

## Introduction

The major goals of nutrition labelling on food packages are to help consumers select healthy foods and to combat widespread, serious nutrient-related diseases. For example, overweight or obesity contributes to the death of 3.4 million people globally [1]. In Thailand, the prevalence of nutrient-related non-communicable disease (NCD) and obesity has increased remarkably over the last two decades to become an urgent national health problem [2]. Diets have become less healthy and physical activity levels have decreased [3]. Thais now consume more processed foods containing high levels of sugar, fat and sodium, and little fibre [4] which are associated with obesity and NCDs [5, 6]. Nutrition labels have been promoted for nearly two decades in Thailand to reduce the consumption of unhealthy foods. But we still know little about how labels are experienced by Thai consumers and whether they are associated with health outcomes.

Previous studies, mostly in North America and Europe, have shown that use of nutrition labels can shift consumers to healthier food consumption patterns [7–9]. Compared to non-users, nutrition label users have lower intakes of fat and cholesterol and higher intakes of fruit, vegetables, and fibre [10, 11]. Even college students, who are not focused on the importance of healthy meals, have healthier diets if they read nutrition labels [12]. Consumers who use serving size information on nutrition labels reported eating 150 kcal less per day than those who were non-label users [13]. Patients with chronic diseases who were advised to use nutrition labels consumed less energy, saturated fat, carbohydrates, and sugar, and more fibre than non-label users [14].

The experiences of nutrition labels among adult Thai consumers at risk of nutrient-related NCDs has not been investigated. Here we report on a cross-sectional study of the associations between nutrition labelling and nutrient-related NCDs building on data from a large existing cohort study of adult Thai open-university students residing nationwide.

## Methods

The Thai Cohort Study (TCS) began in 2005 when distance learning adult students, residing nationwide and enrolled at Sukhothai Thammathirat Open University (STOU), returned a baseline survey and agreed to a longitudinal study of the health-risk transition [15]. Cohort members were of modest means and stayed in their communities but expected to progress because of better education. At baseline they reported their childhood and current environment, occupation, socio-demographic attributes, personal behaviour, transport, well-being, illness, and injury. For this analysis, all 42,750 cohort members who responded to the 8-year follow-up of the TCS in 2013 and were not monks and prisoners (n = 35) were included. Monks and prisoners were excluded because they do not shop for food. Also for analysis of Body Mass Index (BMI) we excluded those classified underweight because interpreting their health status is complex and our NCD focus was on overweight. The TCS participants in 2013 were aged 20 to 96 years, with a majority aged 30 to 45 years. They closely represented the Thai population for sex ratio, median age, religion, regional distribution, and median income [15] and were very similar to the body of distance learning students studying at STOU at baseline in 2005 [16].

Questionnaires covered a broad variety of topics including nutrition label experience, socio-demographic attributes, health behaviour, body size, and health conditions. For analysis, respondents were divided into two age groups: <40 and 40+ years. We noted location of residence (urban or rural), household size (1–2, 3–4, or 5+ people), and monthly income (<10000, 10001–30000, >30000 Baht). The question on exercise asked about the number of sessions per week. The responses led to a metabolically-adjusted physical activity (sessions/week) calculated as “2 × strenuous + moderate + walking” sessions categorized as follows: low activity (< 3 sessions/ week), medium (4–11 sessions/ week), and high (≥12 sessions/week) [17]. Alcohol exposure was grouped as follows: non-drinkers, social drinkers, and heavy drinkers [18]. Smoker categories analyzed were as current smokers or non-smokers.

Three questions about nutrition label experience (read, understand, use) were included in the 2013 questionnaires. Responses to each question were digitized in binary format as follows:

- Read (Yes/No). Derived from “Have you ever seen nutrition labels on food products?” The “Yes” response was “seen and read”; “No” responses were “seen not read” or “unaware”.
- Understand (Good/Not good). Derived from “How well do you understand the information presented on food nutrition labels?” The “Good” responses were “understand fully” or “understand most information”; “Not good” response were “understand some information”, “do not understand information but I know it has potential”, or “do not understand information or its potential”.
- Use (Frequent/ Infrequent). Derived from “How often do you use information from nutrition labels on food products to assist your food purchasing decision?” The “frequent use” responses were “every time” or “often”; “Infrequent use” responses were “sometimes”, “seldom”, or “never”.

In the 2013 TCS survey, BMI was calculated by the formula (BMI = kg/m^2^) and categorized using Asian cut-offs as “normal” (BMI 18.5-<23), “overweight at risk” (BMI 23-<25), and “obese” (BMI ≥25) [19]. Those found to be underweight were excluded (n = 2455, 5.79%) because this category mixes together young people naturally thin, others who seek thinness, and others who are thin due to disease. Self-reported weight and height measures in the study population have been validated [20] and our 8-year longitudinal data revealed rapid increase in overweight and obesity in the cohort [21]. Questions were asked about specific doctor-diagnosed diseases including high blood pressure (HBP) and high blood lipids (HBL). HBP responses have been validated [22].

Data scanning and editing used Thai Scandevet software. Further data editing used SQL and SPSS software. For analysis we used Stata v14. Individuals with missing data were excluded from analysis. Finally, we created a “nutrition label experience” as an exposure dose variable (graded code 1 to code 5) by combining the three experience component measures as follows:

1. “not read” (regardless of use or understanding);
2. “read” but “not good” understanding and “infrequent” use;
3. “read” with “not good” understanding but “frequent” use;
4. “read” with “good” understanding and “infrequent” use;
5. “read” with “good” understanding and “frequent” use.

We noted cross-sectional associations between this label experience measure and the health outcomes and repeated multiple logistic regressions adjusting for potential confounders–the sociodemographic and health covariates. Adjusted Odds Ratios (ORs) are presented with 95% Confidence Intervals.

Ethics approval was obtained from Sukothai Thammathirat Open University Research and Development Institute (protocol 0522/10) and the Australian National University Human research Ethics Committee (protocols 2004/344 and 2009/570). Informed written consent was obtained from all participants.

## Results

### Cohort attributes, nutritional label experience, and health outcome

Of the 42,750 cohort members analysed in 2013 (Table 1), 45.1% were males, 51.3% were aged less than 40 years, and 55.3% reported residing in urban areas. About half reported living in a household of 3–4 members. Personal monthly income was reported to be less than 10,000 Baht (300 USD) by most respondents (59.6%). Almost half (45.2%) reported medium physical activity (4–11 sessions/week). In this cohort, the prevalence of HBP, HBL, and obese body size were 7.6%, 13.9%, and 30.4%, respectively. Our respondents reported that 89% read, 70% understand, and 64% use nutrition labels.

**Table 1:** Frequent nutrition label use by cohort attributes and health outcomes, Thai Cohort Study 2013.

Cohort attributes	n (%)	Proportion of frequent nutrition label users (%) by cohort attributes and health outcomes					
		Self-reported doctor diagnosed health outcomes			Body Mass Index (BMI)^a^		
		Neither high blood pressure nor high blood lipids reported^b^ (n = 33002)	High blood pressure^c^ (n = 3026)	High blood lipids ^d^ (n = 5594)	Normal (n = 18336)	Overweight at risk (n = 9116)	Obese (n = 12005)
Overall	42750 (100)	65.4	60.5	60.6	66.1	64.7	61.5
Sex							
Male	19295 (45.1)	59.6	57.0	55.9	59.0	60.0	57.7
Female	23455 (54.9)	69.7	67.2	66.6	69.8	70.6	66.9
Age (years)							
< 40	21925 (51.3)	63.8	59.2	57.8	64.8	63.2	59.2
≥ 40	20825 (48.7)	67.6	60.8	61.4	67.8	65.8	63.3
Location							
Rural	18913 (44.7)	66.9	60.6	60.7	67.0	66.0	62.9
Urban	23434 (55.3)	64.1	60.5	60.5	65.2	63.6	60.5
Household size							
1–2	8655 (20.5)	64.6	64.8	59.6	65.3	62.9	60.4
3–4	20164 (47.8)	65.2	59.8	60.9	65.9	65.0	61.5
5+	13326 (31.6)	66.3	59.6	60.9	66.7	65.8	62.4
Personal monthly income							
< 10000 Baht	25209 (59.6)	65.1	60.2	60.8	66.1	64.9	60.7
10001–30000 Baht	9234 (21.8)	66.0	57.8	58.8	65.4	64.1	62.4
>30000 Baht	7853 (18.6)	65.8	63.2	61.8	66.9	64.7	62.5
Physical activity^e^							
0–3 sessions/ week	5506 (13.2)	55.4	47.8	51.2	56.5	53.2	51.7
4–11 sessions/ week	18894 (45.2)	64.2	60.4	59.5	64.5	63.4	60.7
≥12 sessions/ week	17433 (41.7)	69.7	65.0	65.6	70.5	69.2	66.1
Alcohol consumption^f^							
Never drinkers	24247 (57.2)	68.0	63.7	63.8	68.4	68.2	64.3
Social drinkers (light)	10367 (24.4)	64.8	58.4	58.1	64.8	63.8	60.8
Heavy drinkers/ social	7798 (18.4)	57.7	55.9	55.0	57.9	57.5	55.8
Current smoker							
No	39292 (92.4)	66.1	61.1	61.4	66.7	65.1	62.5
Yes	3227 (7.6)	56.3	56.5	52.7	56.1	59.8	52.1

^a^ Body Mass Index (Asian cut-off): normal (BMI 18.5-<23), overweight (BMI 23-<25), obese (BMI 25+)

^b^ Column % shows proportion of frequent nutrition label user among cohort members who did not have high blood pressure and/or high blood lipid (calculated separately by each cohort attribute eg sex)

^c^ Column % shows proportion of frequent nutrition label user among cohort members who did not have high blood pressure

^d^ Column % shows proportion of frequent nutrition label user among cohort members who did not have high blood lipid

^e^ Physical activity (sessions/ week) are calculated by "2 × strenuous + moderate + walking exercise sessions"

^f^ Alcohol consumption: 1) Never drink = non-drinker or ex-drinker, 2) Social drink = social with less than 4 glasses/week, 3) Heavy drink = current regular drinker + social with more than 4 glasses/week

Use of nutrition label information s the final goal for health promotion, so in this initial report the analysis is restricted to the associations of label use. In general, respondents with poor health outcomes reported less use of nutrition labels than their healthy counterparts. Females, older age persons, rural residents, and those reporting high physical activity (≥12 sessions/ week) were more frequent label users. Heavy alcohol drinkers and smokers reported less frequent use of nutrition labels, compared to non-drinkers and non-smokers. The lowest proportion (47.8%) of nutrition label users was among a group of participants who had both high blood pressure and low physical activity. The proportion of frequent label use was higher (65.4%) among those without HBP or HBL compared to those who had HBP (60.5%) or HBL (60.6%). Overweight or obese respondents used nutrition labels less than the normal BMI reference group (66.1%).

**Fig 1:**
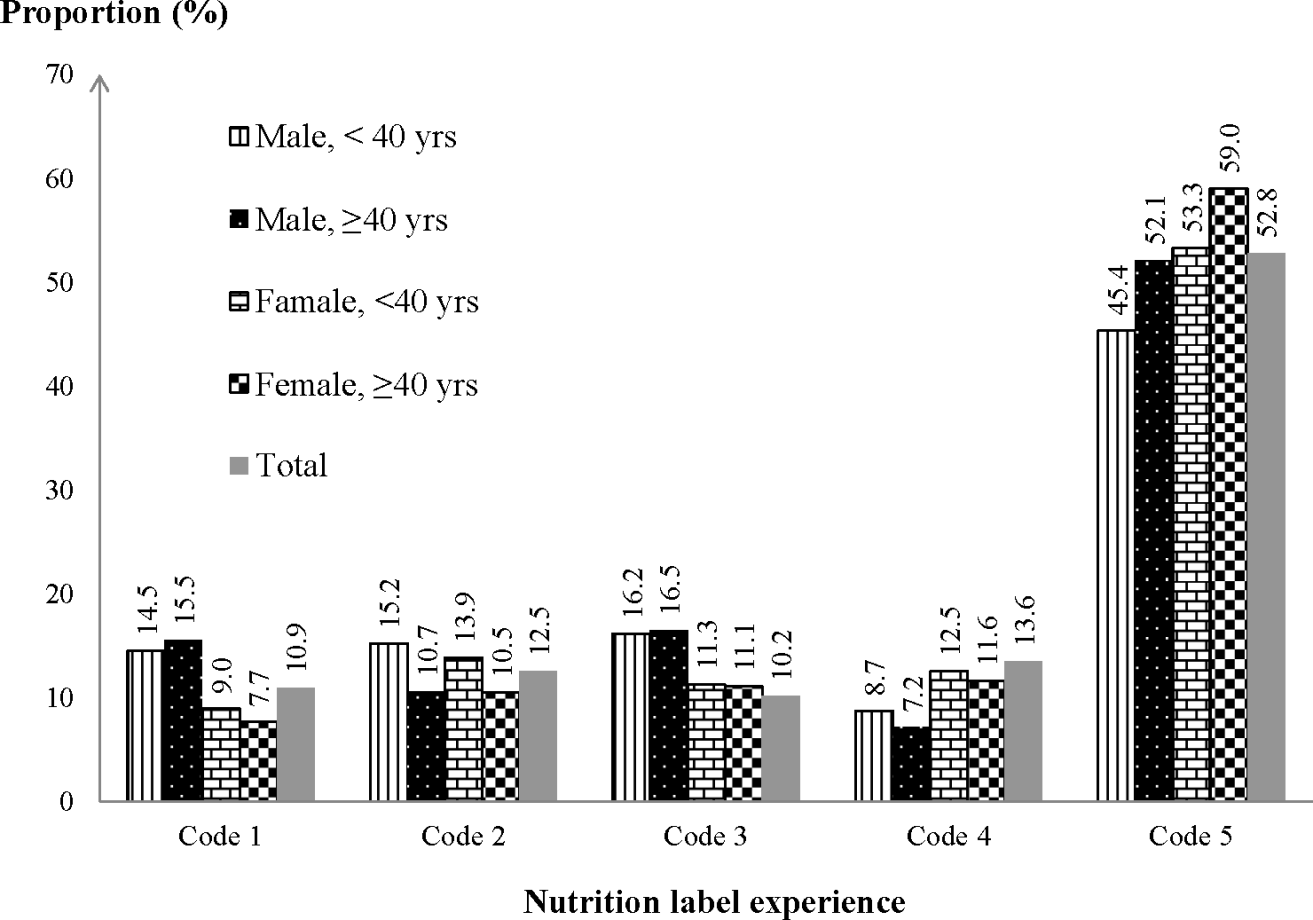
Proportion of cohort participants by age-sex groups in each nutrition label category.

Analysis of the distribution among respondents (by sex and age group) of each category of “exposure doses” of nutrition label experience reveals the uptake of the labels since first appearing in 1998 (Fig 1). The five exposure categories were not evenly distributed. The highest “dose” of nutrition label experience (exposure code 5) was disproportionately frequent with about half of the cohort reporting this level; to compensate, each of the other four “doses” (exposure codes 1–4) were disproportionately less than 20%. Frequent use of nutrition labels without a good understanding of the information (exposure code 3) was reported by 13.6% of respondents. Infrequent use with good understanding (exposure code 4) was reported by 10.2% of respondents. Among the age-sex subgroups the highest proportion for the highest dose of nutrition label (exposure code 5) were older age females (59.0%).

### Association between nutritional label experiences and related health outcomes

We performed multiple logistic regressions exploring the relationship between nutrition label experience (code 1–5) and health outcomes adjusting for an array of covariates (sex, age, location, household size, income, and physical activity, alcohol consumption, and smoker status). We found statistically significant associations between nutrition label experiences (code 5 as reference) and reported HBP, HBL and obesity (Table 2). As the “exposure dose” (codes 4 to 1) of nutrition labelling fell progressively below the code 5 reference there were dose responses with increasing odds for these three adverse health outcomes. Compared to cohort members who read, have good understanding, and frequent use of nutrition labels (i.e. exposure code 5 reference group), those who do not read, do not have good understanding of, and infrequently use labels (exposure code 1) were 1.33, 1.26, and 1.23 times more likely to report having HBP, HBL, or to be obese. Respondents who read and frequently used labels without a good understanding of them (exposure code 3) were more likely to report HBP (OR 1.15; 1.01–1.29), HBL (OR 1.20; 1.10–1.32) but were not overweight at risk, or obese. In the group of overweight at risk there was no statistically significant link to nutrition label experiences.

**Table 2:** Cross-sectional associations between nutrition label experiences and related health conditions adjusted for cohort attributes, Thai Cohort Study 2013.

Cohort characteristics N = 42,750	Adjusted logistic Odd Ratios (AOR, 95% Confidence Interval) ^a^			
	High blood pressure^a^	High blood lipids^a^	Body Mass Index^b^	
			Overweight at risk	Obese
Nutrition label experiences ^c^ (Read—good understand—Frequent use)				
Code (1) No-N/A-N/A	1.33 (1.17–1.51)***	1.26 (1.14–1.39)***	1.06 (0.97–1.16)	1.23 (1.13–1.33)***
Code (2) Yes-No-No	1.21 (1.05–1.38)**	1.27 (1.15–1.40)***	0.92 (0.85–1.01)	1.02 (0.94–1.10)
Code (3) Yes-No-Yes	1.15 (1.01–1.29)*	1.20 (1.10–1.32)***	0.96 (0.89–1.04)	1.04 (0.97–1.12)
Code (4) Yes-Yes-No	1.09 (0.93–1.26)	1.08 (0.96–1.20)	0.96 (0.88–1.05)	0.93 (0.85–1.01)
Code (5) Yes-Yes-Yes	Ref	Ref	Ref	Ref
Sex				
Male	Ref	Ref	Ref	Ref
Female	0.56 (0.51–0.62)***	0.75 (0.70–0.81)***	0.48 (0.45–0.51)***	0.42 (0.40–0.45)***
Age (years)	1.12 (1.11–1.12)***	1.09 (1.08–1.09)***	1.03 (1.03–1.03)***	1.03 (1.03–1.04)***
Location				
Rural	Ref	Ref	Ref	Ref
Urban	1.18 (1.08–1.29)***	1.27 (1.19–1.36)***	0.99 (0.94–1.05)	1.07 (1.02–1.12)**
Household size (no. person)	0.99 (0.97–1.00)	0.97 (0.96–0.99)**	1.02 (1.01–1.04)***	1.03 (1.02–1.04)***
Personal monthly income				
< 10000 Baht	Ref	Ref	Ref	Ref
10001–30000 Baht	1.04 (0.94–1.16)	1.66 (1.54–1.79)***	1.15 (1.08–1.23)***	1.07 (1.01–1.14)*
>30000 Baht	1.08 (0.97–1.19)	1.63 (1.51–1.77)***	1.15 (1.07–1.24)***	1.11 (1.04–1.19)**
Physical activity^d^				
0–3 sessions/ week	1.60 (1.40–1.83)***	1.65 (1.50–1.82)***	1.04 (0.96–1.14)	1.45 (1.35–1.57)***
4–11 sessions/ week	1.26 (1.15–1.38)***	1.31 (1.23–1.41)***	1.04 (0.98–1.10)	1.16 (1.10–1.22)***
≥12 sessions/ week	Ref	Ref	Ref	Ref
Alcohol consumption				
Never drinkers	Ref	Ref	Ref	Ref
Social drinkers (light)	0.76 (0.69–0.85)***	0.93 (0.86–1.01)	1.03 (0.96–1.10)	0.92 (0.87–0.98)**
Heavy drinkers/ social	1.04 (0.93–1.16)	0.98 (0.90–1.08)	1.22 (1.13–1.33)***	1.22 (1.13–1.31)***
Current smoker				
No	Ref	Ref	Ref	Ref
Yes	1.13 (0.98–1.30)	1.03 (0.91–1.15)	0.80 (0.72–0.89)***	0.86 (0.78–0.94)**

^a^ Adjusted Odds Ratio for all factors included in the model

*p<0.1

**p<0.05

***p<0.01

For HBP and HBL, the reference is participants who did not report high blood pressure or high blood lipids as diagnosed by doctors

For BMI, the normal BMI is a reference for both overweight at risk and obese analyses.

^b^ Body Mass Index (Asian cut-off): normal (BMI 18.5-<23), overweight at risk (BMI 23-<25), obese (BMI 25+)

^c^ For analysis, we categorised cohort members reporting every time/often as “nutrition label users”

^d^ Physical activity (sessions/ week) are calculated by "2 × strenuous + moderate + walking exercise sessions"

^e^ Alcohol consumption: 1) Never drink = non-drinker or ex-drinker; 2) Social drink = social with less than 4 glasses/week; 3) Heavy drink = current regular drinker + social with more than 4 glasses/week

Being male and increasing in age strongly associated (p<0.001) with HBP, HBL, overweight, and obesity. Urban participants had higher Odds Ratios of HBP (OR 1.18; 1.08–1.29), HBL (OR 1.27; 1.19–1.36), and obesity (OR1.07; 1.02–1.12). Low physical activity increased risk for HBP (OR 1.60; 1.40–1.83), HBL (OR 1.65; 1.50–1.82), and obesity (OR 1.45; 1.35–1.57). Light alcohol drinkers had low odds ratio of HBP but heavy drinkers were at higher risk of being overweight at risk or obese. Smoking status did not statistically associate with HBP and HBL but was related to decreasing adjusted odds for overweight at risk and obese.

## Discussion

A Thai Food and Drug Administration public health intervention has supported nutrition labelling of food for the last 18 years and this program has reached a large part of the population. Our cohort resides nationwide and over half of the participants were aware, understood and used the information. We found cross-sectional associations between respondents not reading nutrition labels (i.e. unexposed to the intervention) and a higher occurrence of nutrient-related health outcomes (HBP, HBL, obesity) in Thai adults. This study also found that self-reported HBP, HBL, obesity were associated with sex, age, urban residence, and low physical activity.

Associations between nutrition label experiences and adverse health conditions have never been explored in previous Thai studies partly due to the relatively low number of disease cases available in the population. Our large study found a lower proportion of frequent nutrition label users among participants with nutrient-related health outcomes which contrasts with the widespread assumption that people with adverse health conditions will use nutrition labels more [23]. Our previous qualitative study found that people who had developed concerns about their health said they were likely to adopt the use of nutrition labels [24]. It is possible that nutrition label experience was low among those who became diseased and subsequently rose but did not reach the level of non-disease counterparts. The current study is not likely to capture this dynamic transition as we do not have longitudinal measurement of nutrition label experience.

In this study, we found similar factors associated with nutrient-related health outcomes and obesity as reported in other studies. Being male and older age were associated with higher risk of HBP, HBL, and obesity. Other studies also show prevalence of HBP was higher among men and older age groups [25–27] while women are less affected than men [28]. Also we found women were more likely to be experienced nutrition label users (read, good understanding, and frequent use).

Recent reports show that urbanisation associates with hypertension and obesity [29, 30]. We provide more evidence that higher HBP, HBL, and obesity link to urban residence. Light alcohol consumption has sometimes been associated with health benefits [31, 32] but our results are mixed. Social drinkers had lower adjusted odds of HBP but heavy drinkers were more likely to report overweight and obesity. The lower adjusted odds of being overweight and obese among smokers in the study reflects the finding that nicotine reduces appetite leading to lower body weight [33].

Our study has some limitations. First, this is a cross-sectional study, so it cannot be used to causally link the impact of nutrition labels to health outcomes due to the possibility of reverse causation. However, this could be explored in the future when new longitudinal data become available. Second, our data on nutrition label experiences was based on respondent self-report and their understanding of information on nutrition labels. It was not feasible to use a questionnaire to ascertain the determinants of their levels of understanding. Nevertheless, our study adds to existing limited evidence on the patterns of nutrition label use and health in a large nationwide adult sample.

We have demonstrated that the population enrolled in 2005 were similar to the general Thai adult population for age, sex, region, religion, and ethnicity [15]. In addition the enrolled cohort represented well the student body at STOU and cohort attrition at follow up in 2013 was minimized by an array of successful methods [16, 34]. The attrition that did occur mostly effected young unmarried urban males and this group were relatively unaffected by the health outcomes measured and reported [35]. Also our cohort consists of Open University adults who contain a higher proportion of high school completers than the average for the Thai population. It is likely that a lower fraction of the general Thai population will be nutrition label users than in our more educated sample. But cohort members are of modest socio-economic means and remain embedded in their communities. By their educational attainment they point the way to the Thais of tomorrow.

We found that nutrition label non-users are statistically more likely to have HBP, HBL, and obesity. Also participants with these adverse health conditions and with low physical activity levels were considerably less likely to use nutrition labels. It is possible that people with lower physical activity levels are less willing, or able, to address their health conditions. More research with this group would help explore this in greater depth.

## Conclusions

The use of nutrition labels has the potential to help people prevent obesity and nutrient-related NCDs. The Thai government could disseminate more widely information about the value of nutrition labels to assist with appropriate food choices.

## Supporting information

S1 File 2013 TCS survey questionnaire (original in Thai). (PDF)

S2 File 2013 TCS survey questionnaire (translated in English). (PDF)
