# Supplementary material for: Nutrition label experience, obesity, high blood pressure, and high blood lipids in a cohort of 42,750 Thai adults
Source: PLoS One. 2017 Dec 13;12(12):e0189574. doi: 10.1371/journal.pone.0189574 (PMC5728572; doi:10.1371/journal.pone.0189574)

แบบสอบถาม

โครงการวิจัยสุขภาพ ปี 2556

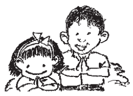

เรียน สมาชิกโครงการวิจัยสุขภาพทุกท่าน

ความก้าวหน้าของโครงการวิจัยสุขภาพที่ท่านมีส่วนสำคัญยิ่งต่อความสำเร็จนั้น ได้ก้าวมาสู่ขั้นการดำเนินงานวิจัยทางสุขภาพอย่างเต็มรูปแบบ ข้อมูลที่ท่านได้ตอบแบบสอบถามกลับมา โครงการฯ ได้นำมาวิเคราะห์ปัจจัยที่ส่งผลต่อสุขภาพ นั้น

บัดนี้ เป็นช่วงเวลาการติดตามสภาวะสุขภาพของสมาชิกโครงการฯ ข้อมูลที่ท่านตอบกลับมาจะเป็นประโยชน์อย่างยิ่งต่อการสร้างองค์ความรู้ด้านสุขภาพในบริบทของสังคมไทย และโครงการฯ จะได้ดำเนินการตามแนวจริยธรรมของการวิจัยอย่างเคร่งครัด ข้อมูลรายบุคคลจะถูกเก็บเป็นความลับ สำหรับชื่อและที่อยู่ทางโครงการฯ จะใช้สำหรับการติดต่อกับท่านเท่านั้น โดยตระหนักดีว่าท่านได้ให้ความร่วมมือตอบแบบสอบถามด้วยความสมัครใจ อย่างไรก็ตาม หากท่านไม่มีความประสงค์จะเป็นสมาชิกของโครงการฯ ท่านสามารถแจ้งให้โครงการฯ ทราบทุกเมื่อ

หาก**ท่านคือบุคคลที่ปรากฏชื่อตามเอกสารด้านบน**นี้ และยินดีให้ความร่วมมือกับโครงการวิจัยสุขภาพ มหาวิทยาลัยสุโขทัยธรรมาธิราช โปรดลงชื่อในช่องว่างข้างล่างแล้วกรุณาตอบแบบสอบถาม ส่งกลับมาในซองที่แนบมาพร้อมนี้ โดยไม่ต้องติดแสตมป์

(ลงชื่อ)... วันที่........./........../........

(นาย/นาง/นางสาว ....)

หากมีข้อสงสัย สอบถามรายละเอียดเพิ่มเติมได้ที่หมายเลขโทรศัพท์ 02-5047780 ในเวลาราชการ ขอขอบคุณทุกท่านมา ณ โอกาสนี้

(รองศาสตราจารย์ ดร. สำอาง สืบสมาน)
ผู้อำนวยการศูนย์วิจัยและสร้างเสริมสุขภาวะคนไทย

**หน้านี้จะได้รับจัดเก็บเป็น
ความลับแยกออกจากส่วนอื่น**

ความร่วมมือตอบแบบสอบถามของสมาชิกทุกท่าน คือสิ่งสำคัญสูงสุดต่อความสำเร็จของโครงการวิจัยสุขภาพ โดยที่สามารถติดต่อกับท่านได้ หากท่านเปลี่ยนแปลงชื่อ ที่อยู่ หรือหมายเลขโทรศัพท์ โปรดแจ้งให้ทางโครงการฯ ทราบตามแบบฟอร์มข้างล่างนี้ จะขอบคุณยิ่ง

ท่านมีการ เปลี่ยนแปลง **ชื่อ-สกุล ที่อยู่ เบอร์โทรศัพท์ ไปจากหน้าปกแบบสอบถาม** หรือไม่ โปรด **กาเครื่องหมาย ✕ ลงช่องสี่เหลี่ยม ❑ โดยใช้ปากกาสีน้ำเงินหรือดำ**

❑ **ไม่มีการเปลี่ยนแปลง** ทั้งชื่อ - สกุล ที่อยู่ และเบอร์โทรศัพท์ → **โปรดข้ามไปอ่านคำชี้แจงหน้าถัดไป**

❑ **มีการเปลี่ยนแปลงข้อมูล** ในรายการต่อไปนี้ ❑ **ชื่อ - สกุล** ❑ **ที่อยู่** ❑ **เบอร์โทรศัพท์**
โดยมีรายละเอียดที่เปลี่ยนแปลงดังนี้

ชื่อ.. นามสกุล ....
ที่อยู่ : เลขที่.. หมู่บ้าน .... ซอย ....
ถนน.. ตำบล/แขวง .... อำเภอ/เขต ....
จังหวัด... รหัสไปรษณีย์ ☐☐☐☐☐
เบอร์โทรศัพท์บ้าน.. โทรศัพท์ที่ทำงาน..
โทรศัพท์มือถือ.. e-mail...

บุคคลอื่นที่สามารถติดต่อได้ (กรณีที่ติดต่อท่านไม่ได้)

ชื่อ.. นามสกุล ....
ที่อยู่ : เลขที่.. หมู่บ้าน .... ซอย ....
ถนน.. ตำบล/แขวง .... อำเภอ/เขต ....
จังหวัด... รหัสไปรษณีย์ ....
เบอร์โทรศัพท์บ้าน.. โทรศัพท์ที่ทำงาน..
โทรศัพท์มือถือ..

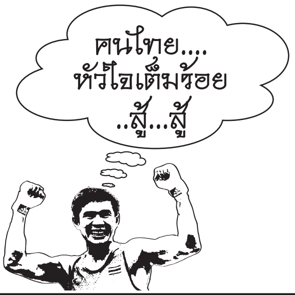

กรุณาจดเลขหมายรหัสสมาชิก TCSID จากหน้าปกเก็บไว้ใช้อ้างอิงในอนาคต เพื่อความสะดวกของท่านในการติดต่อกับโครงการวิจัยสุขภาพ

หน้านี้จะได้รับจัดเก็บเป็นความลับแยกออกจากส่วนอื่น

คำชี้แจง โปรดใช้**ปากกาสีน้ำเงินหรือดำ กาเครื่องหมาย × ลงช่องสี่เหลี่ยม** ☐ หน้าตัวเลือกที่ต้องการดังรูป ☒ โดยเลือกเพียงคำตอบเดียว **ยกเว้น**ข้อที่มีระบุว่า "เลือกได้**มากกว่า** 1 คำตอบ" ส่วนคำถามที่ให้เขียนคำตอบเป็นตัวเลขนั้น โปรดใส่ตัวเลขลงภายในช่อง ☐ ช่องละหนึ่งตัวเลข เช่น **24** ให้แยกตัวเลขลงในช่องดังนี้ **| 2 | 4 |**

A ข้อมูลเกี่ยวกับตัวท่านและการงานของท่าน

A1 เพศ ☐ ชาย ☐ หญิง

A2 วัน เดือน ปีเกิดของท่าน (ตามบัตรประจำตัวประชาชน)

☐☐ / ☐☐ / ☐☐☐☐

วันที่ เดือน ปี พ.ศ.ที่เกิด

(เช่น | 1 | 5 | / | 0 | 1 | / | 2 | 5 | 1 | 3 | คือเกิด 15 มกราคม 2513)

A3 บ้านที่ท่านพักอาศัยใน__ปัจจุบัน__อยู่ที่ไหน

☐ ในชนบท ☐ ในเมือง

A4 ท่านอาศัยอยู่บ้านหลังนี้มานานเท่าไร ☐☐ **ปี**

(เช่น ถ้าอยู่มานาน 3 ปี โปรดใส่ตัวเลข | 0 | 3 | ปี)

A5 บ้านของท่านอยู่ห่างจากสถานที่ต่อไปนี้เป็นระยะทางประมาณกี่กิโลเมตร (กม.)

ระยะทาง / สถานที่	น้อยกว่า 5 กม.	5-10 กม.	11-20 กม.	ไกลกว่า 20 กม. ขึ้นไป
ซูเปอร์มาร์เก็ต, มินิมาร์ท	☐	☐	☐	☐
ตู้กดเงิน ATM	☐	☐	☐	☐
โรงพยาบาล	☐	☐	☐	☐
ไปรษณีย์	☐	☐	☐	☐
ที่ว่าการอำเภอ/เขต	☐	☐	☐	☐
โรงเรียน	☐	☐	☐	☐
สี่แยกไฟแดง	☐	☐	☐	☐
ตลาดสด	☐	☐	☐	☐
ป้ายรถประจำทาง	☐	☐	☐	☐
วินมอเตอร์ไซค์	☐	☐	☐	☐
ร้านอินเตอร์เน็ต	☐	☐	☐	☐

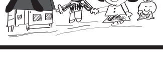

A6 ในช่วง __5 ปีที่ผ่านมา__ ท่านคิดว่าท้องถิ่นที่ท่านอาศัยอยู่ในปัจจุบันเป็นชุมชนเมืองมากขึ้น ใช่หรือไม่

☐ ใช่ ☐ ไม่ใช่ ☐ ไม่แน่ใจ

A7 บ้านของท่านมีอยู่กี่คน ☐☐ **คน** *(รวมทั้งตัวท่าน)*

(เช่น ถ้าอยู่ 3 คน โปรดใส่ ตัวเลข | 0 | 3 |)

A8 ปัจจุบันท่านทำงานที่มีรายได้ใช่หรือไม่

☐ ใช่ ☐ ไม่ใช่ → ข้ามไปตอบข้อ **A14**

A9 ปัจจุบันท่านทำงานที่สร้างรายได้ประมาณ__สัปดาห์__ละกี่ชั่วโมง ☐☐☐ **ชั่วโมง/สัปดาห์**

A10 ท่านรู้สึกมั่นคงในอนาคตการทำงานหรืออาชีพที่ทำอยู่ขณะนี้เพียงใด

☐ ไม่มั่นคงเลย ☐ มั่นคงเล็กน้อย

☐ มั่นคง ☐ มั่นคงมาก

A11 งานหลักที่ท่านทำมีลักษณะตรงกับข้อใดมากที่สุด

(เลือกเพียง 1 คำตอบ)

☐ ผู้บริหารระดับอาวุโส ☐ ผู้บริหารระดับกลาง

☐ งานวิชาชีพ (เช่น นักบัญชี แพทย์ นักวิชาการ)

☐ ช่างชำนาญงาน (เช่น งานฝีมือ ช่างไม้ ช่างผม)

☐ เจ้าหน้าที่ในสำนักงาน/บริษัท

☐ เกษตร/ประมง/เลี้ยงสัตว์

☐ ทำงานโรงงาน

☐ งานขั้นพื้นฐานใช้แรงเบาๆ (เช่น ส่งเอกสาร)

☐ งานขั้นพื้นฐานที่ใช้แรงมาก (เช่น งานก่อสร้าง)

☐ อื่นๆ โปรดระบุ...............................

A12 ในช่วง <u>12 เดือนที่ผ่านมา</u> ท่านประสบปัญหาจากความร้อน/อากาศร้อน<u>ในขณะทำงาน</u> จนทำให้ท่านรู้สึกไม่สบายตัว เนื่องจากสาเหตุใดบ่อยที่สุด
(เลือกเพียง 1 คำตอบ)

- ☐ ไม่มีปัญหาเรื่องความร้อนรบกวนในที่ทำงาน
- ☐ ความร้อนจากการทำงานกลางแจ้ง
- ☐ ความร้อนจากเครื่องจักร หรือกระบวนการผลิต
- ☐ ความร้อนจากการทำงานที่ต้องใช้ยานพาหนะ
- ☐ ความร้อนจากการทำงานในอาคารที่อบอ้าว
- ☐ อื่นๆ โปรดระบุ..............................

A13 ในช่วง <u>12 เดือนที่ผ่านมา</u> เมื่อท่านมีปัญหาความร้อนในขณะทำงาน *จากข้อA12* **ทำให้ท่านมีอาการในข้อใดต่อไปนี้** *(เลือกได้มากกว่า 1 คำตอบ)*

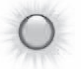

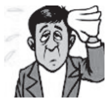

- ☐ ไม่มีอาการใดๆ
- ☐ รู้สึกไม่สบายตัวเท่านั้น
- ☐ มีผด / ผื่นคัน
- ☐ ปวดศีรษะ / ปวดขมับ
- ☐ คลื่นไส้ / อาเจียน
- ☐ รู้สึกขาดน้ำอย่างรุนแรง / กระหายน้ำมาก
- ☐ วิงเวียน / หน้ามืด / เป็นลม (แบบมีเหงื่อออก)
- ☐ เป็นลมแดด - หมดสติ โดยร่างกายมีอุณหภูมิสูง ผิวหนังร้อนและแห้ง (แบบไม่มีเหงื่อ)
- ☐ ปวดกล้ามเนื้อ / เป็นตะคริว
- ☐ ความดันต่ำ

A14 ท่านมีรายได้ประมาณ<u>เดือนละ</u>เท่าไร

- ☐ น้อยกว่าหรือ= 3,000 บาท
- ☐ 3,001 - 7,000 บาท
- ☐ 7,001 - 10,000 บาท
- ☐ 10,001 - 20,000 บาท
- ☐ 20,001 - 30,000 บาท
- ☐ มากกว่า 30,000 บาทขึ้นไป

A15 ครอบครัวของท่านมีรายได้ประมาณ<u>เดือนละ</u>เท่าไร

- ☐ น้อยกว่าหรือ= 3,000 บาท
- ☐ 3,001 - 7,000 บาท
- ☐ 7,001 - 10,000 บาท
- ☐ 10,001 - 20,000 บาท
- ☐ 20,001 - 30,000 บาท
- ☐ มากกว่า 30,000 บาทขึ้นไป

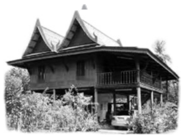

A16 การศึกษาสูงสุดของท่าน *(ไม่รวมที่กำลังศึกษาอยู่ขณะนี้)*

- ☐ ม.3 หรือเทียบเท่า
- ☐ ม.6 / ปวช. หรือเทียบเท่า
- ☐ ป.วิชาชีพเทคนิค/ชั้นสูง/อนุปริญญา
- ☐ ปริญญาตรีหรือสูงกว่า

A17 สถานภาพสมรสของท่านในปัจจุบัน *(เลือกเพียง 1 คำตอบ)*

- ☐ แต่งงานครั้งแรก
- ☐ แต่งงานครั้งที่ 2 หรือมากกว่า

} ข้ามไปตอบข้อ **A19**

- ☐ แยกทางกัน/เลิกกันแล้ว (แต่ยังไม่ได้หย่า)
- ☐ หย่า ☐ หม้าย (คู่สมรสเสียชีวิต)
- ☐ ไม่เคยแต่งงาน

A18 ถ้าปัจจุบันยังไม่แต่งงาน ท่านมีแฟน(คนรัก)หรือไม่

- ☐ มีและอยู่ด้วยกัน ☐ มี แต่ไม่ได้อยู่ด้วยกัน
- ☐ ไม่มี

A19 ท่านสูงเท่าไร ☐☐☐ **เซ็นติเมตร** *(ไม่สวมรองเท้า)*

A20 ปัจจุบันท่านมีน้ำหนักเท่าไร ☐☐☐ **กิโลกรัม**

(เช่น ท่านมีน้ำหนัก 62 กิโลกรัม โปรดใส่ตัวเลข 0 6 2 *)*

A21 ท่านต้องดูแลสมาชิกในครอบครัว หรือคนรู้จักที่ป่วยเรื้อรัง/ทุพพลภาพ/ชราภาพหรือไม่

- ☐ ดูแล ☐ ไม่ได้ดูแล → ข้ามไปตอบข้อ **B1**

A22 ท่านดูแลบุคคลที่ป่วยเรื้อรัง/ทุพพลภาพ/ชราภาพสัปดาห์ละกี่ชั่วโมง ☐☐☐ **ชั่วโมง/สัปดาห์**

A23 ท่านดูแลบุคคลดังกล่าวข้างต้น มาแล้วกี่ปี ☐☐ **ปี**

A24 ท่านช่วยดูแลบุคคลดังกล่าวข้างต้นอย่างไรบ้าง

(เลือกได้มากกว่า 1 คำตอบ)

- ☐ ช่วยเตรียมหรือป้อนอาหาร
- ☐ ช่วยอาบน้ำ ☐ ช่วยแต่งตัว
- ☐ ช่วยขยับตัว เคลื่อนย้าย
- ☐ ช่วยพาไปวัด/ร่วมพิธีทางศาสนา
- ☐ ช่วยซื้อของกินของใช้
- ☐ ช่วยทำให้คลายเครียด
- ☐ ช่วยด้านความทรงจำ เช่น อธิบายเรื่องราวต่างๆ
- ☐ ช่วยด้านการเงิน ☐ อื่นๆ

B สุขภาวะโดยรวมของท่าน

B1 ในช่วง <u>4 สัปดาห์ที่ผ่านมา</u> โดยทั่วไปท่านประเมินสุขภาพของท่านว่าอย่างไร

☐ ดีที่สุด ☐ ดีมาก ☐ ดี
☐ พอใช้ ☐ แย่ ☐ แย่มาก

B2 ในช่วง <u>4 สัปดาห์ที่ผ่านมา</u> ปัญหาสุขภาพกายจำกัดกิจกรรมทางกายตามปกติของท่าน *(เช่น การเดิน หรือการขึ้นลงบันได)* **มากน้อยแค่ไหน**

☐ ไม่เลย ☐ น้อยมาก
☐ พอสมควร ☐ ค่อนข้างมาก
☐ ไม่สามารถทำกิจกรรมทางกายต่างๆ ได้

B3 ในช่วง <u>4 สัปดาห์ที่ผ่านมา</u> ท่านมีความลำบากในการทำงานประจำวัน ทั้งงานในบ้านและนอกบ้านที่เป็นผลมาจากสุขภาพทางกายของท่าน มากน้อยแค่ไหน

☐ ไม่มีเลย ☐ มีน้อยมาก
☐ มีพอสมควร ☐ มีค่อนข้างมาก
☐ ไม่สามารถทำงานประจำวันได้

B4 ในช่วง <u>4 สัปดาห์ที่ผ่านมา</u> ท่านมีความเจ็บปวด<u>ทางร่างกาย</u> มากน้อยแค่ไหน

☐ ไม่มีเลย ☐ น้อยมาก ☐ น้อย
☐ ปานกลาง ☐ รุนแรง ☐ รุนแรงมาก

B5 ในช่วง <u>4 สัปดาห์ที่ผ่านมา</u> ท่านมีความรู้สึกกระฉับกระเฉง มากน้อยแค่ไหน

☐ มีมาก ☐ มีค่อนข้างมาก ☐ มีบ้าง
☐ มีเล็กน้อย ☐ ไม่มีเลย

B6 ในช่วง <u>4 สัปดาห์ที่ผ่านมา</u> สุขภาพทางกาย หรือปัญหาด้านอารมณ์ของท่าน จำกัดกิจกรรมทางสังคมตามปกติของท่านที่มีกับครอบครัว หรือเพื่อน มากน้อยแค่ไหน

☐ ไม่เลย ☐ น้อยมาก
☐ พอสมควร ☐ ค่อนข้างมาก
☐ ไม่สามารถทำกิจกรรมทางสังคมต่างๆ ได้

B7 ในช่วง <u>4 สัปดาห์ที่ผ่านมา</u> ปัญหาด้านอารมณ์ *(เช่น รู้สึกกังวล ซึมเศร้า หรือหงุดหงิด)* **รบกวนท่านมากน้อยแค่ไหน**

☐ ไม่เลย ☐ เล็กน้อย ☐ ปานกลาง
☐ ค่อนข้างมาก ☐ มากที่สุด

B8 ในช่วง <u>4 สัปดาห์ที่ผ่านมา</u> ปัญหาส่วนตัวหรือปัญหาด้านอารมณ์ ทำให้ท่าน<u>ไม่สามารถ</u>ทำงาน เรียนหนังสือ หรือทำกิจกรรมประจำวันอื่นๆ ของท่านได้ตามปกติ มากน้อยเพียงใด

☐ ไม่เลย ☐ น้อยมาก
☐ พอสมควร ☐ ค่อนข้างมาก
☐ ไม่สามารถทำกิจกรรมประจำวันต่างๆ ได้

B9 ในช่วง <u>4 สัปดาห์ที่ผ่านมา</u> ภาวะสุขภาพของท่านเป็นอุปสรรคต่อการทำกิจกรรมต่อไปนี้ มากน้อยเพียงใด

**อุปสรรคต่อกิจกรรม** ต่อไปนี้	ไม่มีเลย	มีเล็กน้อย	มีมาก
การขึ้นบันได 1 ชั้น	☐	☐	☐
การเดิน 100 เมตร	☐	☐	☐
การคุกเข่า เอี้ยวตัว โน้มตัว	☐	☐	☐
การใส่เสื้อผ้าแต่งตัว	☐	☐	☐

B10 ในช่วง <u>4 สัปดาห์ที่ผ่านมา</u> ท่านปวดหลังส่วนล่าง *(บริเวณที่แสดงในรูป)* **หรือไม่**

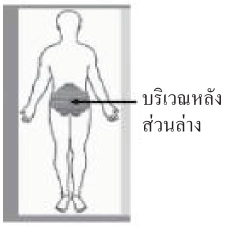

☐ ใช่
☐ ไม่ใช่ → **ข้ามไปตอบข้อ B12**

B11 <u>ถ้าใช่</u> อาการปวดนั้นทำให้ท่าน<u>ไม่สามารถ</u>ทำงาน หรือ<u>ไม่สามารถ</u>ทำกิจกรรมประจำวันอื่นๆ ได้มากกว่า 1 วันขึ้นไป ใช่หรือไม่

☐ ใช่ ☐ ไม่ใช่

B12 ผู้ใหญ่มีฟันแท้ทั้งสิ้น 32 ซี่ ท่านมีฟันแท้จำนวนกี่ซี่

☐ ไม่มีเลย ☐ 1-5 ซี่

☐ 6-19 ซี่ ☐ 20 ซี่หรือมากกว่า

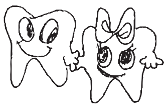

B13 ปัจจุบัน ฟันแท้หรือฟันปลอมของท่าน ทำให้ท่านมีอาการต่อไปนี้หรือไม่ *(เลือกได้มากกว่า 1 คำตอบ)*

☐ ไม่สะดวกเวลาพูด ☐ กลืนไม่สะดวก

☐ เคี้ยวไม่สะดวก ☐ ขาดความมั่นใจเมื่อเข้าสังคม

☐ รู้สึกเจ็บปวด ☐ ไม่มีอาการดังกล่าว

C ชีวิตของท่าน

C1 ท่านได้รับการสนับสนุนจากกลุ่มทางสังคมมากน้อยแค่ไหน *(โปรดใส่เครื่องหมาย x ลงในช่องที่ตรงกับความคิดเห็นของท่าน)*

	น้อยมาก	ค่อนข้างน้อย	ค่อนข้างมาก	มากที่สุด	ไม่มีบุคคลดังกล่าว
ครอบครัวของท่าน	☐	☐	☐	☐	☐
เพื่อนบ้าน	☐	☐	☐	☐	☐
เพื่อน	☐	☐	☐	☐	☐
นายจ้าง/หัวหน้า	☐	☐	☐	☐	☐

C2 ในช่วง <u>4 สัปดาห์ที่ผ่านมา</u> ท่านมีความรู้สึกต่อไปนี้บ่อยแค่ไหน *(โปรดกา X ลงในช่องที่ตรงกับความรู้สึกของท่านในแต่ละข้อ)*

ความรู้สึก *(ในช่วง 4 สัปดาห์ที่ผ่านมา)*	ตลอดเวลา	เกือบตลอดเวลา	บางเวลา	น้อยครั้ง	ไม่เคยเลย
รู้สึกเศร้ามากจนไม่มีอะไรช่วยให้ดีขึ้นได้	☐	☐	☐	☐	☐
รู้สึกกังวล	☐	☐	☐	☐	☐
รู้สึกกระวนกระวาย	☐	☐	☐	☐	☐
รู้สึกหมดหวัง	☐	☐	☐	☐	☐
รู้สึกว่าต้องใช้ความพยายามในการทำทุกสิ่งทุกอย่าง	☐	☐	☐	☐	☐
รู้สึกไร้ค่า	☐	☐	☐	☐	☐
รู้สึกมีความสุข	☐	☐	☐	☐	☐

C3 ท่านรู้สึกระมัดระวังหรือกังวลเรื่องน้ำหนักตัวเมื่ออยู่ต่อหน้าคนอื่นบ่อยแค่ไหน

☐ บ่อยครั้ง ☐ บางครั้ง ☐ ไม่เลย

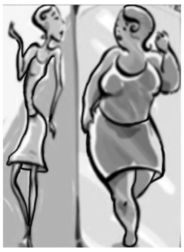

C4 เกี่ยวกับรูปร่างของท่านในปัจจุบัน ท่านต้องการทำอย่างไร

☐ ลดน้ำหนัก ☐ เพิ่มน้ำหนัก ☐ ให้น้ำหนักคงเดิมอยู่เช่นนี้

C5 ในช่วง <u>12 เดือนที่ผ่านมา</u> ท่านควบคุมอาหารเพื่อจุดประสงค์ใด ☐ เพิ่มน้ำหนัก ☐ ลดน้ำหนัก ☐ ไม่เคยควบคุม

C6 ท่านมีปัญหาบ่อยแค่ไหนในการควบคุมการกินอาหาร ☐ บ่อยครั้ง ☐ บางครั้ง ☐ ไม่มีปัญหา

C7 เมื่อคิดถึงชีวิตและสภาพแวดล้อมของท่าน ท่านมีความพอใจกับสิ่งต่อไปนี้ในระดับใด *(โปรดใส่เครื่องหมาย X ลงในช่องระดับ 0 ⟶ 10 คะแนน ที่ตรงกับความคิดเห็นของท่าน)*

ความพอใจต่อชีวิตและสภาพแวดล้อม	ไม่พอใจอย่างยิ่ง ⟵⟶ พอใจที่สุด										
	0	1	2	3	4	5	6	7	8	9	10
มาตรฐานการดำรงชีวิต	☐	☐	☐	☐	☐	☐	☐	☐	☐	☐	☐
ความรู้สึกปลอดภัย	☐	☐	☐	☐	☐	☐	☐	☐	☐	☐	☐
ความรู้สึกเป็นส่วนหนึ่งของชุมชน	☐	☐	☐	☐	☐	☐	☐	☐	☐	☐	☐
ชีวิตโดยรวมของท่าน	☐	☐	☐	☐	☐	☐	☐	☐	☐	☐	☐
เวลาว่างส่วนตัว	☐	☐	☐	☐	☐	☐	☐	☐	☐	☐	☐

C8 โดยทั่วไป ท่านรู้สึกว่าสามารถวางใจผู้อื่นได้เพียงใด

☐ สามารถวางใจได้เกือบทุกคน ☐ ต้องระมัดระวังผู้อื่นตลอดเวลา

C9 <u>น้ำท่วมเมื่อปี 2554</u> ท่านได้รับผลกระทบทางกายภาพมากน้อยแค่ไหน *(บ้านเรือน ทรัพย์สิน ฯลฯ)*

☐ มากที่สุด ☐ ค่อนข้างมาก ☐ เล็กน้อย ☐ ไม่เลย

C10 <u>น้ำท่วมเมื่อปี 2554</u> ท่านได้รับผลกระทบทางจิตใจมากน้อยแค่ไหน

☐ มากที่สุด ☐ ค่อนข้างมาก ☐ เล็กน้อย ☐ ไม่เลย

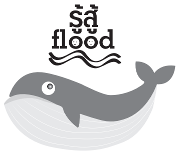

C11 โดยภาพรวม <u>ในปี 2555</u> ท่านได้รับผลกระทบจากน้ำท่วมมากน้อยแค่ไหน เมื่อเทียบกับปี 2554

☐ มากกว่าเดิม ☐ น้อยกว่าเดิม ☐ เท่าเดิม ☐ ไม่ได้รับผลกระทบ

D อาหารและกิจกรรมการเคลื่อนไหวร่างกาย

D1 ท่านรับประทานผักจำนวนกี่ส่วนต่อวัน ☐☐ **ส่วนต่อวัน** เช่น ถ้ากิน 3 ส่วน ใส่ตัวเลข | 0 | 3 |

(เช่น ผัก 1 ส่วน = ผักปรุงสุก ครึ่งถ้วยตวง หรือผักดิบ 1 ถ้วยตวง)

D2 ท่านรับประทานผลไม้จำนวนกี่ส่วนต่อวัน ☐☐ **ส่วนต่อวัน** เช่น ถ้ากิน 5 ส่วน ใส่ตัวเลข | 0 | 5 |

(เช่น กล้วย 1 ลูก = 1 ส่วน หรือ มะละกอ 1 ชิ้น 5-6 คำ = 1 ส่วน)

D3 ตามปกติเวลากินอาหาร ท่านเติมน้ำปลาเฉลี่ย กี่ช้อนชาต่อวัน ☐☐ **ช้อนชาต่อวัน** *ถ้าไม่เติมเลย ใส่ตัวเลข* | 0 | 0 |

(ช้อนชา คือ ช้อนกาแฟเล็กๆ)

D4 ตามปกติเวลากินอาหาร/เครื่องดื่ม ท่านเติมน้ำตาลเฉลี่ย กี่ช้อนชาต่อวัน ☐☐ **ช้อนชาต่อวัน** *ถ้าไม่เติมเลย ใส่* | 0 | 0 |

D5 ท่านเคยเห็น "ฉลากโภชนาการ" หรือไม่

☐ เคยเห็นและเคยอ่านแล้ว ☐ เคยเห็นแต่ยังไม่เคยอ่าน ☐ ไม่เคยรู้จักเลย

D6 ท่านใช้ ฉลากโภชนาการ ประกอบการตัดสินใจซื้อผลิตภัณฑ์อาหาร บ่อยครั้งแค่ไหน

☐ ทุกครั้งที่ซื้อ ☐ บ่อยครั้ง ☐ บางครั้ง

☐ นานๆครั้ง ☐ ไม่เคยเลย

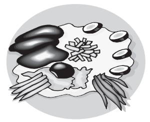

D7 ท่านเข้าใจข้อมูลบนฉลากโภชนาการดีเพียงใด

☐ เข้าใจดีอย่างยิ่ง ☐ เข้าใจเป็นส่วนใหญ่ ☐ เข้าใจบางส่วน

☐ ไม่เข้าใจ แต่รู้ว่ามีประโยชน์ ☐ ไม่เข้าใจและไม่รู้ว่ามีประโยชน์

D8 ท่านอยากให้มีฉลากโภชนาการครอบคลุมอาหารชนิดต่างๆ มากขึ้นหรือไม่

☐ ใช่ ☐ ไม่ใช่ ☐ ไม่ทราบ

D9 โดยเฉลี่ยแล้ว ท่านรับประทานอาหารต่อไปนี้ บ่อยครั้งแค่ไหน*(กาเครื่องหมาย x ลงช่องที่ตรงตามที่ท่านรับประทานจริง)*

	ไม่เคย/น้อยกว่าเดือนละครั้ง	เดือนละ 1-3 ครั้ง	สัปดาห์ละ 1-2 ครั้ง	สัปดาห์ละ 3-6 ครั้ง	วันละครั้งหรือมากกว่า
อาหาร/ขนมหวานที่ประกอบด้วยกะทิ	☐	☐	☐	☐	☐
อาหารประเภททอด	☐	☐	☐	☐	☐
อาหารกึ่งสำเร็จรูป เช่น บะหมี่ซอง	☐	☐	☐	☐	☐
อาหารหมักดอง (ดิบ) เช่น แหนม ปลาร้า	☐	☐	☐	☐	☐
ผักผลไม้ดอง	☐	☐	☐	☐	☐
ข้าวจ้าวหรือข้าวเหนียวขัดขาว	☐	☐	☐	☐	☐
ข้าวกล้อง/หรือข้าวขาวผสมข้าวกล้อง	☐	☐	☐	☐	☐
ปลา/ผลิตภัณฑ์จากปลา	☐	☐	☐	☐	☐
น้ำอัดลม เช่น โค้ก เป๊ปซี่ ฯลฯ	☐	☐	☐	☐	☐
เครื่องดื่มผสมน้ำตาล เช่น ชาเขียว กาแฟเย็น น้ำสมุนไพร	☐	☐	☐	☐	☐
นม เช่น นมสด นมกล่อง นมผง ฯลฯ	☐	☐	☐	☐	☐
ผลิตภัณฑ์อาหารเสริม เช่น วิตามิน	☐	☐	☐	☐	☐
อาหารจานด่วนแบบตะวันตก เช่น แฮมเบอร์เกอร์ พิซซ่า	☐	☐	☐	☐	☐
ขนมแบบตะวันตก เช่น โดนัท คุ้กกี้ เค้ก	☐	☐	☐	☐	☐

D10 ในวันธรรมดาที่ผ่านมา (ไม่ใช่วันหยุด) **ท่านรับประทานอาหารต่างๆ กี่ครั้ง** ☐☐ **ครั้งต่อวัน** *(รวมมื้อหลักและมื้อว่าง)*

D11 ในช่วง 7 วันที่ผ่านมา ท่านรับประทานอาหารมื้อหลักโดยลำพังกี่ครั้ง ☐☐ **ครั้งต่อวัน**

D12 เมื่อท่านกินอาหารตามลำพัง ท่านกินมากขึ้นหรือน้อยลง เมื่อเทียบกับการกินร่วมกับคนอื่นๆ

☐ มากขึ้น ☐ น้อยลง ☐ เท่าเดิม

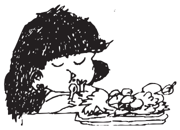

D13 โดยปกติ ท่านใช้เวลาในการเคลื่อนไหวร่างกายดังต่อไปนี้ ประมาณกี่ครั้งในหนึ่งสัปดาห์

(เช่น ถ้าเคลื่อนไหวร่างกาย 3 ครั้งต่อสัปดาห์ โปรดใส่ตัวเลข 0 3 *ถ้าไม่ได้ทำเลย โปรดใส่ตัวเลข* 0 0 *)*

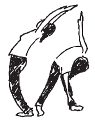

**เดินอย่างต่อเนื่อง <u>อย่างน้อย 10 นาที</u>**<br>(เช่น เดินเพื่อทำงาน เดินพักผ่อน เดินออกกำลังกาย หรือเดินเพื่อไปถึงที่ใดที่หนึ่ง)	☐☐ ครั้งต่อสัปดาห์
**การเคลื่อนไหวร่างกายที่ใช้แรงมาก <u>นานกว่า 20 นาทีขึ้นไป</u>**<br>(ทำให้หายใจแรงและเร็ว เช่น แอโรบิค กีฬาที่ใช้แรงมาก ขี่จักรยาน วิ่ง)	☐☐ ครั้งต่อสัปดาห์
**การเคลื่อนไหวร่างกายที่ใช้แรงระดับปานกลาง <u>นานกว่า 20 นาทีขึ้นไป</u>**<br>(เช่น การสังสรรค์ เล่นเทนนิส กอล์ฟ ว่ายน้ำช้าๆ ทำงานบ้าน หรืองานอื่นๆ)	☐☐ ครั้งต่อสัปดาห์

D14 ท่านทำงานบ้าน เช่น ทำความสะอาด หรือทำสวน บ่อยครั้งเพียงใด

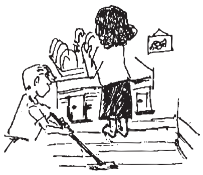

- ☐ น้อยมากหรือไม่เคยเลย
- ☐ 1 - 3 ครั้ง / เดือน
- ☐ 1 - 2 ครั้ง / สัปดาห์
- ☐ 3 - 4 ครั้ง / สัปดาห์
- ☐ เกือบทุกวันหรือทุกวัน

D15 โดยปกติ ในหนึ่งวัน (24 ชั่วโมง) ท่านใช้เวลาในการทำสิ่งเหล่านี้ประมาณวันละกี่ชั่วโมง

กิจกรรม	ระยะเวลา
**ยืนทุกกรณี** (เช่น ยืนทำงาน ยืนคุย ฯลฯ)	☐☐ ชั่วโมงต่อวัน
**นั่งทุกกรณี** (เช่น นั่งอ่านหนังสือ ขีดเขียน นั่งพักผ่อน นั่งคิด รวมเวลาที่นั่งดูโทรทัศน์ หรือนั่งทำงาน/เล่นเกมคอมพิวเตอร์)	☐☐ ชั่วโมงต่อวัน
**นอน** (หากนอนกลางวันเป็นประจำให้นับรวมด้วย)	☐☐ ชั่วโมงต่อวัน
**นั่งเฉพาะกรณีดูโทรทัศน์และ/หรือนั่งเล่นคอมพิวเตอร์/นั่งเล่นเกม**	☐☐ ชั่วโมงต่อวัน

E การบาดเจ็บ

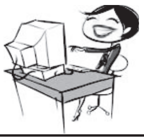

การบาดเจ็บ-ที่เกี่ยวข้องกับการจราจร

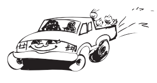

E1 ในช่วง <u>12 เดือนที่ผ่านมา</u> ท่านเคยได้รับบาดเจ็บจากอุบัติเหตุที่เกี่ยวข้องกับการจราจร จำนวนกี่ครั้ง

- ☐ ไม่เคยได้รับบาดเจ็บ → ข้ามไปตอบข้อ **E7**
- ☐ 1 ครั้ง
- ☐ 2 ครั้ง
- ☐ 3 ครั้ง
- ☐ 4 ครั้งขึ้นไป

E2 การบาดเจ็บที่เกี่ยวกับการจราจรครั้ง<u>ที่เจ็บหนักที่สุด</u>นั้น ท่านต้องเข้ารับการรักษาพยาบาลหรือไม่

- ☐ ใช่
- ☐ ไม่ใช่

E3 การบาดเจ็บครั้งนั้นมีผลทำให้ท่าน<u>ไม่สามารถ</u>ใช้ชีวิตได้ตามปกติตั้งแต่ 1 วันขึ้นไปหรือไม่

- ☐ ใช่
- ☐ ไม่ใช่

E4 การบาดเจ็บครั้งนั้นท่านมีบทบาทอย่างไร

- ☐ เป็นผู้ขับขี่
- ☐ เป็นผู้โดยสาร
- ☐ เป็นผู้สัญจร → ข้ามไปตอบข้อ **E6**

E5 การบาดเจ็บครั้งนั้นยานพาหนะที่ท่านขับขี่/โดยสาร คือ

- ☐ รถจักรยาน
- ☐ รถมอเตอร์ไซค์
- ☐ รถโดยสาร/รถตู้/รถทัวร์
- ☐ รถยนต์/รถปิ๊กอัพ
- ☐ อื่นๆ เช่น รถไฟ เรือ

E6 คู่กรณี ที่ทำให้ท่านได้รับบาดเจ็บครั้งนั้น คือ

- ❑ รถจักรยาน
- ❑ รถมอเตอร์ไซค์
- ❑ รถโดยสาร/รถตู้/รถทัวร์
- ❑ รถยนต์/รถปิ๊กอัพ
- ❑ ยานพาหนะอื่นๆ เช่น รถไฟ รถอีแต๋น เรือ
- ❑ ผู้สัญจร
- ❑ สัตว์ เช่น สุนัข
- ❑ สิ่งอื่นที่ไม่ใช่ยานพาหนะ เช่น ชนต้นไม้ กำแพง ผิวถนน

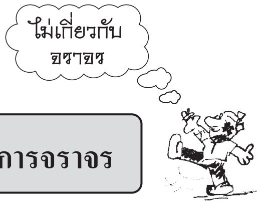

การบาดเจ็บ - ที่ไม่เกี่ยวข้องกับการจราจร

E7 ในช่วง 12 เดือนที่ผ่านมา ท่านเคยได้รับการบาดเจ็บที่ไม่เกี่ยวข้องกับการจราจร จำนวนกี่ครั้ง

- ❑ ไม่เคยได้รับบาดเจ็บ → ข้ามไปตอบข้อ **F1**
- ❑ 1 ครั้ง
- ❑ 2 ครั้ง
- ❑ 3 ครั้ง
- ❑ 4 ครั้งขึ้นไป

E8 การบาดเจ็บที่ไม่เกี่ยวข้องกับการจราจรครั้งที่เจ็บหนักที่สุดนั้น ท่านต้องเข้ารับการรักษาพยาบาลหรือไม่

- ❑ ใช่
- ❑ ไม่ใช่

E9 การบาดเจ็บครั้งนั้นมีผลทำให้ไม่สามารถใช้ชีวิตได้ตามปกติตั้งแต่ 1 วันขึ้นไปหรือไม่

- ❑ ใช่
- ❑ ไม่ใช่

E10 การบาดเจ็บครั้งที่เจ็บหนักที่สุดนั้น เกิดขึ้นอย่างไร

- ❑ ถูกทำร้าย (เช่น ถูกชก ผลัก เตะ)
- ❑ ถูกกระแทก/ของตกใส่หรืออื่นๆ
- ❑ ถูกของมีคมบาด/แทง
- ❑ ถูกยิง
- ❑ พลัดตกหรือหกล้มเอง
- ❑ ยกของหนัก
- ❑ แผลไฟไหม้หรือน้ำร้อนลวก
- ❑ จมน้ำ
- ❑ ถูกแมลง/สัตว์/กัดหรือต่อย
- ❑ ถูกสารพิษ
- ❑ สำลัก
- ❑ อื่น ๆ

E11 การบาดเจ็บที่ไม่เกี่ยวกับการจราจรครั้งที่เจ็บหนักที่สุดนั้น เกิดขึ้นที่ไหน

- ❑ ที่พักอาศัย
- ❑ สถานที่เล่นกีฬาหรือออกกำลังกาย
- ❑ สถานที่ทำงาน (ด้านเกษตรกรรม เช่น ไร่ นา)
- ❑ สถานที่ทำงานอื่นๆ (ที่ไม่เกี่ยวกับเกษตรกรรม)
- ❑ อื่นๆ

E12 การบาดเจ็บที่ไม่เกี่ยวกับการจราจรครั้งนั้น มีอาการแบบใด *(เลือกได้มากกว่า 1 คำตอบ)*

- ❑ กระดูกหัก แตก ร้าว
- ❑ เคล็ด ขัดยอกหรือข้อเคลื่อน
- ❑ แผลจากของมีคม แผลถูกกัด หรือแผลเปิด
- ❑ ฟกช้ำหรือบาดเจ็บบริเวณผิวหนัง
- ❑ แผลไฟไหม้/น้ำร้อนลวก
- ❑ แผลปูด/บวมจากการถูกอัด/กระแทก
- ❑ อวัยวะภายในร่างกายได้รับบาดเจ็บ
- ❑ อื่นๆ

E13 การบาดเจ็บที่ไม่เกี่ยวกับการจราจรครั้งนั้น เกิดขึ้นอย่างไร *(เลือกเพียง 1 คำตอบ)*

- ❑ ไม่ได้ตั้งใจ เป็นอุบัติเหตุ
- ❑ ตั้งใจกระทำโดยบุคคลอื่น
- ❑ ตั้งใจกระทำ (ไม่มีบุคคลอื่นเกี่ยวข้อง)

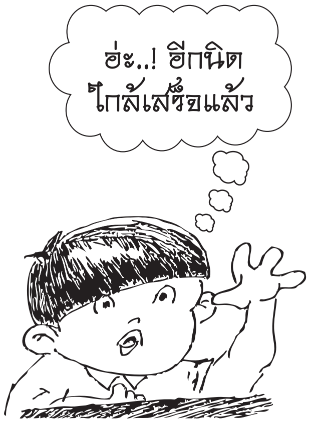

F ประวัติเกี่ยวกับสุขภาพของท่าน

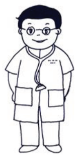

F1 ท่านเคยได้รับการยืนยันอย่างชัดเจน จากแพทย์ว่าท่านเป็นโรคใดต่อไปนี้บ้าง

รายการ	เป็น	แพทย์บอกว่าท่านเสี่ยงที่จะเป็น	ไม่เป็น
เบาหวาน	☐	☐	☐
คอเลสเตอรอลสูงหรือไขมันในเลือดสูง	☐	☐	☐
ความดันโลหิตสูง	☐	☐	☐
โรคหัวใจขาดเลือด	☐	☐	☐
โรคหลอดเลือดในสมอง (Stroke)	☐	☐	☐
มะเร็งตับ	☐	☐	☐
มะเร็งปอด	☐	☐	☐
มะเร็งกระเพาะอาหาร	☐	☐	☐
มะเร็งลำไส้	☐	☐	☐
มะเร็งเต้านม	☐	☐	☐
มะเร็งอวัยวะอื่นๆ	☐	☐	☐
โรคเกี่ยวกับไต	☐	☐	☐
โรคอื่นๆ(ระบุ)...	☐	☐	☐

F2 ปัจจุบันท่านมีหลักประกันสุขภาพประเภทใดบ้าง และคุ้มครองมานานกี่ปี *(เลือกได้มากกว่า 1 คำตอบ)*

(ถ้าปัจจุบันคุ้มครองมาแล้วน้อยกว่า 1 ปี โปรดใส่ตัวเลข 0 1 *)*

ประเภทของหลักประกันสุขภาพ	ระยะเวลาที่คุ้มครองมาแล้ว
☐ ไม่มีสวัสดิการใดๆ	
☐ สวัสดิการข้าราชการ	☐☐ ปี
☐ สวัสดิการพนักงาน/ลูกจ้าง	☐☐ ปี
☐ ประกันสุขภาพของเอกชน	☐☐ ปี
☐ ประกันสังคม	☐☐ ปี
☐ ประกันสุขภาพถ้วนหน้า	☐☐ ปี
☐ อื่นๆ...	☐☐ ปี

F3 ในช่วง 12 เดือน ที่ผ่านมา ท่านเข้ารับบริการสุขภาพกี่ครั้ง จากสถานพยาบาลใดบ้าง *(เลือกได้มากกว่า 1 คำตอบ)*

สถานพยาบาล	จำนวนครั้ง (ในช่วง 12 เดือนที่ผ่านมา) *เช่น ใช้บริการปีละครั้งใส่* 0 1 *ครั้ง*
สถานีอนามัย	☐☐ ครั้ง
โรงพยาบาลชุมชน	☐☐ ครั้ง
คลินิกเอกชน	☐☐ ครั้ง
โรงพยาบาลของรัฐ	☐☐ ครั้ง
โรงพยาบาลของเอกชน	☐☐ ครั้ง
แพทย์แผนไทย	☐☐ ครั้ง
ร้านขายยา	☐☐ ครั้ง
อื่นๆ..........................	☐☐ ครั้ง

G บุหรี่ แอลกอฮอล์ และการเดินทาง

G1 ปัจจุบันท่านสูบบุหรี่หรือไม่

☐ ไม่สูบ ☐ สูบ โดยสูบวันละ ☐☐ มวน

G2 การดื่มแอลกอฮอล์ของท่านในปัจจุบันเป็นแบบใด

☐ ไม่เคยดื่มเลย ☐ เคยดื่ม แต่เลิกแล้ว

☐ ดื่มเฉพาะเวลาเข้าสังคม ประมาณ ☐☐ แก้ว/สัปดาห์

☐ ดื่มเป็นประจำ ประมาณ ☐☐ แก้ว/วัน

G3 ในช่วง 12 เดือนที่ผ่านมา ท่านเคยขับขี่ยานพาหนะหลังดื่มแอลกอฮอล์ตั้งแต่ 3 แก้วขึ้นไปหรือไม่

☐ เคย ☐ ไม่เคย

☐ ปกติไม่ได้ขับขี่ยานพาหนะ

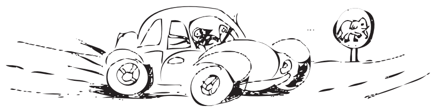

G4 ในช่วง 12 เดือนที่ผ่านมา ท่านกระทำสิ่งต่อไปนี้บ่อยครั้งเพียงใด ในการเดินทาง

	**ทำประจำ**	**ทำบางครั้ง**	**ไม่เคยทำ**	**ไม่เข้าข่าย เนื่องจาก**	
คาดเข็มขัดนิรภัยเมื่อนั่งรถยนต์เบาะหน้า	☐	☐	☐	☐	รถไม่มีเข็มขัดนิรภัย/ไม่นั่งรถยนต์เบาะหน้า
คาดเข็มขัดนิรภัยเมื่อนั่งรถยนต์เบาะหลัง	☐	☐	☐	☐	รถไม่มีเข็มขัดนิรภัย/ไม่นั่งรถยนต์เบาะหลัง
นั่ง/ยืนบริเวณบันไดท้ายรถสองแถว	☐	☐	☐	☐	ไม่ใช้รถสองแถว
นั่งในกระบะท้ายไม่มีหลังคา	☐	☐	☐	☐	ไม่นั่งในกระบะท้าย
สวมหมวกนิรภัยขณะขับขี่/ซ้อนมอเตอร์ไซค์	☐	☐	☐	☐	ไม่ใช้รถมอเตอร์ไซค์
ขับขี่/ซ้อนมอเตอร์ไซค์สามคนหรือมากกว่า	☐	☐	☐	☐	ไม่ใช้รถมอเตอร์ไซค์

H คำถามเกี่ยวกับการให้กำเนิดและคุมกำเนิด *(เฉพาะสมาชิกสตรี ของโครงการวิจัยสุขภาพเท่านั้น)*

*(สมาชิก**ชายไม่ต้องตอบ**ข้อต่อไปนี้)*

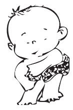

H1 ท่านเคยให้กำเนิดบุตรมาแล้วทั้งหมดกี่คน ☐☐ **คน** *(กรณีโสด หรือไม่เคยให้กำเนิดบุตร โปรดใส่ตัวเลข* 0 0 *)*

H2 โปรดระบุการคุมกำเนิดที่ท่านเคยใช้ในอดีตและปัจจุบัน *(ถ้าใช้มาน้อยกว่า 1 ปี โปรดใส่ตัวเลข* 0 0 *)*

**ท่านเคยใช้การคุมกำเนิดด้วยวิธีต่อไปนี้หรือไม่**		**อายุที่เริ่มใช้ครั้งแรก**	**อายุที่ใช้ครั้งล่าสุด** *(อายุปัจจุบันถ้ายังใช้อยู่)*	**ใช้มานานกี่ปีแล้ว** *(ไม่รวมช่วงที่ไม่ใช้)*
ยาเม็ดคุมกำเนิด	☐ ไม่ใช่<br>☐ ใช่ →	☐☐ ปี	☐☐ ปี	☐☐ ปี
ยาฉีดคุมกำเนิดทุก 3 เดือน (เดโป-โปรวีร่า)	☐ ไม่ใช่<br>☐ ใช่ →	☐☐ ปี	☐☐ ปี	☐☐ ปี
ยาคุมกำเนิดแบบฝังใต้ผิวหนัง (มีผลในการคุม 3-5 ปี)	☐ ไม่ใช่<br>☐ ใช่ →	☐☐ ปี	☐☐ ปี	☐☐ ปี

ขอขอบคุณในความร่วมมืออย่างดียิ่ง

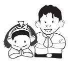

Supplement: S1 File — (PDF) [file pone.0189574.s001.pdf]
