# Supplementary material for: Nutrition label experience, obesity, high blood pressure, and high blood lipids in a cohort of 42,750 Thai adults
Source: PLoS One. 2017 Dec 13;12(12):e0189574. doi: 10.1371/journal.pone.0189574 (PMC5728572; doi:10.1371/journal.pone.0189574)

Questionnaire

Thai Health Research Project 2013

Universal codes:

-7 = "Multiple response". It is assigned to data item where **program** detected that **there are more than one checkbox** being checked for any multiple choice questions.

-9 = "Missing value". It is assigned to data item where **program** detected that the data image is **blank.**

addsend

Dear Thai Cohort Study members

TCSID ☐☐☐☐☐ tcsid

With all of your much valued help our Thai Health Research Project has achieved substantial success. Analysing the information you have provided in your questionnaire responses has helped us understand better the risks to Thai peoples' health.

It is now time for us to follow up again on the health of our members. The further information you provide here will help us assess the health of Thai people and be of benefit to all of Thai society. The project follows strict ethical standards and all of your personal information will be held in the strictest confidence. Your name and address will be kept separately from your other data and will only be used to contact you. Your continued involvement in this project is completely voluntary, and if you wish to withdraw at any time please inform us.

If **you are the person whose name appears on the document above** and you are willing to continue participating in this health research project based at Sukhothai Thammathirat Open University please write your name and sign this form below. When you have completed the questionnaire please return it in the envelope included here. You do not need to attach a stamp.

(Name)..sign1.......... Date.........dsign/........./........

(Mr/Mrs/Miss ....sign2..)

If you have any doubts or concerns or need more information on the project please contact us on 02-5047780 during business hours. Thanks and regards.

(Associate Professor Sam-ang Seubsman)

Director Thai Health Research Project

This page will be separated and treated as confidential

Your assistance in filling out this form is very important for the success of our research project. We need to know if your name, address or other details have changed from those shown on the front of the envelope.

Have you changed your **name-surname, address or telephone number from those shown on the front cover of this questionnaire?**

Please **place a cross ✕ in the appropriate boxes** ☐. **Please use a blue or black pen**

addch

☐ 1 **Have not changed** name-surname, address or telephone number → **Please go to the instructions at the top of the next page**

☐ 2 **There has been a change** to my personal information as follows

☐ **Name-surname** addch1 ☐ **Address** addch2 ☐ **Telephone number** addch3

Please give details below

First Name........name1........ Family Name ....sname1........

Family Name........add1n1........ Moo Ban ....add1n2........ Soi ....add1n3........

Road........add1n4........ Tambol/Kwang ....add1n5........ District/Khet ....add1n6`........

Province........add1n7........ Postcode ☐☐☐☐☐ add1zip

Home Tel........add1htel........ Office Tel........add1wtel........

Mobile........add1mtel........ Email........email........

Other contact person (if we cannot contact you)

First Name........name2........ Family Name ....sname2........

Family Name........add2n1........ Moo Ban ....add2n2........ Soi ....add2n3........

Road........add2n4........ Tambol/Kwang ....add2n5........ District/Khet ....add2n6........

Province........add2n7........ Postcode ☐☐☐☐☐ add2zip

Home Tel........add2htel........ Office Tel........add2wtel........

Mobile........add2mtel........

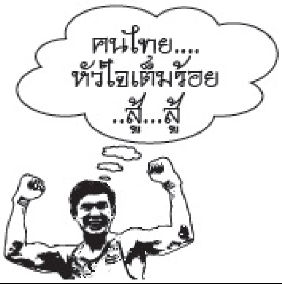

Please keep a record of your member code (TCSID) from the front of the envelope to use as a reference in any future communication with the Thai Health Risk Transition Study

This page will be separated and treated as confidential

Instructions: Use **a blue or black pen to put a cross** × in the ☐ next to the selected choice to get to this image ☒. Select one answer **except** when told **"**You may choose **more than** one answer." For numeric answers, write number(s) **clearly** in the box(es) - one number per box. eg [2][4]

A Information on you and your work

A1 Sex a1 ☐1 Male ☐2 Female

A2 When were you born *(according to your Citizen ID Card)*

[ ][ ] / [ ][ ] / [ ][ ][ ][ ]

a2bd a2bm a2by

(eg [1][5]/[0][1]/[2][5][1][3] *if born 15 January 2513 please put)*

A3 Where is your current residence located?

a3 ☐1 Countryside ☐2 City/town

A4 How long have you lived at your current residence? a3

a4 [ ][ ] **years** *(eg if you have lived there 3 years please write* [0][3] *Years)*

A5 How far in kilometres is it from your current residence to each of the following places? (kms)

Place \ Distance	Less than 5 kms	5-10 kms.	11-20 kms	More than 20 kms
Supermarket/ minimart a5n1	☐1	☐2	☐3	☐4
ATM a5n2	☐1	☐2	☐3	☐4
Hospital a5n3	☐1	☐2	☐3	☐4
Post office a5n4	☐1	☐2	☐3	☐4
District office a5n5	☐1	☐2	☐3	☐4
School a5n6	☐1	☐2	☐3	☐4
Traffic light a5n7	☐1	☐2	☐3	☐4
Fresh market a5n8	☐1	☐2	☐3	☐4
Bus stop a5n9	☐1	☐2	☐3	☐4
Motorcycle taxi stand a5n10	☐1	☐2	☐3	☐4
Internet café a5n11	☐1	☐2	☐3	☐4

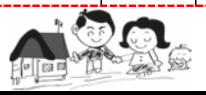

A6 In the past 5 years, has the area where you currently live become more urbanized?

a6 ☐1 Yes ☐2 No ☐3 Unsure

A7 How many people live at your current residence?

a7 [ ][ ] **people** *(including you)*

(eg if there are 3 people please put [0][3] *)*

A8 At present do you have any paid employment?

a8 ☐1 Yes ☐2 No → Go to question **A14**

A9 At present how many hours of paid work do you do per week? [ ][ ][ ] **hours/week**

a9

A10 How secure do you feel about your job or career future in your current occupation?

a10 ☐1 Not at all secure ☐2 Moderately secure ☐3 Secure ☐4 Extremely secure

A11 Which of the following best describes your primary occupation? *(Please choose only one answer)*

a11
- ☐1 Senior manager
- ☐2 Middle manager
- ☐3 Professional (eg accountant, doctor, academic)
- ☐4 Skilled worker (eg carpenters, hairdresser, craftsman)
- ☐5 Office assistant
- ☐6 Agricultural or fisheries worker
- ☐7 Factory or assembly worker
- ☐8 Elementary worker (non-physical) (eg courier)
- ☐9 Elementary worker (physical) (eg construction)
- ☐10 Other, please explain.....a11x.....................

A12 In the past 12 months, have you experienced uncomfortably hot temperatures in your workplace arising primarily from one of the following causes?

(Please choose only one answer)

a12
- 1 I am not bothered by high temperatures at work
- 2 Heat from working outdoors
- 3 Heat from machinery or production processes
- 4 Heat from working in a vehicle
- 5 Heat from work in a stuffy/poorly ventilated building
- 6 Other, please explain.......................... a12x

A13 In the past 12 months, when you experienced the workplace heat exposure described *in question A12*, **what was your reaction?** *(You may choose more than one answer)*

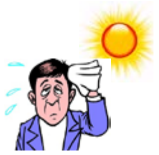

- a13n1 ☐ No reaction
- a13n2 ☐ Mild discomfort
- a13n3 ☐ Prickly heat/ heat rash
- a13n4 ☐ Headache/migraine
- a13n5 ☐ Nausea or vomiting
- a13n6 ☐ Severe dehydration
- a13n7 ☐ Dizziness/ fainting with sweating
- a13n8 ☐ Heat stroke (disorientation /elevated body temp but no sweating)
- a13n9 ☐ Muscle cramps
- a13n10 ☐ Low blood pressure

A14 What is your personal average monthly income?

a14
- 1 ≤3,000 baht
- 2 3,001 - 7,000 baht
- 3 7,001 - 10,000 baht
- 4 10,001 - 20,000 baht
- 5 20,001 - 30,000 baht
- 6 > 30,000 baht

A15 What is your household's average monthly income?

a15
- 1 ≤3,000 baht
- 2 3,001 - 7,000 baht
- 3 7,001 - 10,000 baht
- 4 10,001 - 20,000 baht
- 5 20,001 - 30,000 baht
- 6 > 30,000 baht

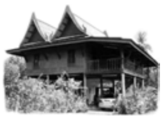

A16 What is your highest level of education *(not including any current studies)*?

a16
- 1 Junior high school or equivalent
- 2 Completed high school or equivalent
- 3 Post-high school diploma or certificate
- 4 Bachelor or higher university degree

A17 What is your current marital status? *(Please choose only one answer)*

a17
- 1 First marriage
- 2 Remarried } Go to question **A19**
- 3 Separated (but not divorced)
- 4 Divorced 5 Widowed 6 Never married

A18 If not currently married, do you have a partner?

a18
- 1 Yes and we live together 2 Yes but don't live
- 3 Don't have a partner

A19 How tall are you? ☐☐☐ a19 **cms** *(without shoes)*

A20 What is your weight now? ☐☐☐ a20 **kgs**

(eg if your weight is 62 kgs write 0 6 2*)*

A21 Do you currently care for a chronically ill/disabled/ or frail family member or other person you know?

a21
- 1 Yes
- 2 No → Go to question **B1**

A22 How many hours per week do you care for this chronically ill/disabled/or frail person?

a22 ☐☐☐ **hours/ week**

A23 How many years have you cared for the person mentioned above? a23 ☐☐ **years**

A24 What type/s of care do you provide to the person mentioned above?

(You may choose more than one answer)

- a24n1 ☐ Help prepare food or eat
- a24n2 ☐ Help bathe a24n3 ☐ Help getting dressed
- a24n4 ☐ Mobility (moving the person)
- a24n5 ☐ Help going to temples/attending religious activities
- a24n6 ☐ Shopping and/or providing daily food
- a24n7 ☐ Emotional support/cheering up
- a24n8 ☐ Cognitive care (helping to understand)
- a24n9 ☐ Financial support ☐ Other a24n10

B Your general health

B1 Overall how would you rate your health in the <u>past 4 weeks</u>?

b1
1 Excellent 2 Very good 3 Good
4 Fair 5 Poor 6 Very poor

B2 During the <u>past 4 weeks</u>, how much did physical health problems limit your usual physical activities *(such as walking or climbing stairs)*?

b2
1 Not at all 2 Very little
3 Some 4 Quite a lot
5 Could not do physical activities

B3 During the <u>past 4 weeks</u>, how much difficulty did you have doing your daily work, both at home and away from home, because of your physical health?

b3
1 None at all 2 A little bit
3 Some 4 Quite a lot
5 Could not do daily work

B4 How much <u>bodily</u> pain have you had during the <u>past 4 weeks</u>?

b4
1 None 2 Very mild 3 Mild
4 Moderate 5 Severe 6 Very severe

B5 During the <u>past 4 weeks</u>, how much energy did you have?

b5
1 Very much 2 Quite a lot 3 Some
4 A little 5 None

B6 During the <u>past 4 weeks</u>, how much did your physical health or emotional problems limit your usual social activities with family or friends?

b6
1 Not at all 2 Very little
3 Somewhat 4 Quite a lot
5 Could not do social activities

B7 During the <u>past 4 weeks</u>, how much have you been bothered by emotional problems *(such as feeling anxious, depressed or irritable)***?**

b7
1 Not at all 2 Slightly 3 Moderately
4 Quite a lot 5 Extremely

B8 During the <u>past 4 weeks</u>, how much did personal or emotional problems <u>keep you from</u> doing your usual work, school or other daily activities?

b8
1 Not at all 2 Very little
3 Somewhat 4 Quite a lot
5 Could not do daily activities

B9 In the <u>past 4 weeks</u>, to what extent has your health limited you in any of the following physical activities?

Limitation for the following physical activities		Not at all	A little	A lot
Climbing a flight of stairs	b9n1	1	2	3
Walking one hundred metre	b9n2	1	2	3
Bending, kneeling or stoopi	b9n3	1	2	3
Dressing yourself	b9n4	1	2	3

B10 In the <u>past 4 weeks</u>, have you had pain in your low back *(in the area shown in the diagram)***?**

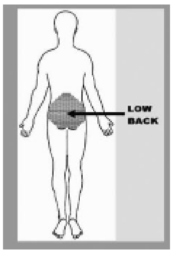

b10
1 Yes
2 No → Go to question **B12**

B11 <u>If yes</u>, was this pain bad enough <u>to limit</u> your usual activities or change your daily routine for more than one day?

b11
1 Yes 2 No

B12 Adults can have up to 32 natural teeth. How many of your own teeth do you have?

b12
1 None
2 1-5 teeth
3 6-19 teeth
4 20 teeth or more

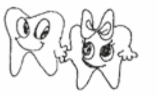

B13 Do your teeth or dentures currently cause you….

(You may choose more than one answer)

- b13n1 Discomfort speaking
- b13n2 Discomfort swallowing
- b13n3 Discomfort chewing
- b13n4 Loss of social confidence
- b13n5 Pain
- b13n6 None of these

C Your Life

C1 How much support do you feel you get from each of the following groups. *(please place a cross ✕ in the box which best applies to you for each question)*

	Very little	A little	Quite a lot	A lot	Not applicable
Family c1n1	1	2	3	4	5
Neighbours c1n2	1	2	3	4	5
Friends c1n3	1	2	3	4	5
Employee/ supervisor c1n4	1	2	3	4	5

C2 In the past 4 weeks how much of the time did you feel…?*(please place a cross ✕ in the box which fits best for each question)*

Your feelings *(in the past 4 weeks)*	All of the time	Most of the time	Some of the time	A little of the time	None of the time
…so sad nothing could cheer you up? c2n1	1	2	3	4	5
…nervous? c2n2	1	2	3	4	5
…restless or fidgety? c2n3	1	2	3	4	5
…hopeless? c2n4	1	2	3	4	5
…everything was an effort? c2n5	1	2	3	4	5
…worthless? c2n6	1	2	3	4	5
…happy? c2n7	1	2	3	4	5

C3 How often do you feel self-conscious or worried in the company of others because of your weight?

c3
1 Often
2 Sometimes
3 Never

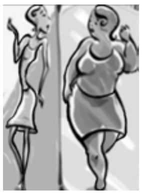

C4 Regarding your current body size: Do you feel you need to

c4
1 Gain weight
2 Lose weight
3 Stay the same

C5 In the past 12 months, have you modified your diet to

c5
1 Gain weight
2 Lose weight
3 Did not modify diet

C6 How often do you have trouble controlling your food intake?

c6
1 Often
2 Sometimes
3 Never

C7 Thinking about your own life and personal circumstances, how satisfied are you with…

(Cross box on 0 ⟶ 10 scale that fits best for each question)

Satisfaction with:		Completely Dissatisfied ⟵									⟶ Completely Satisfied
		0	1	2	3	4	5	6	7	8	9	10
…your standard of living?	c7n1	☐	☐	☐	☐	☐	☐	☐	☐	☐	☐	☐
…how safe you feel?	c7n2	☐	☐	☐	☐	☐	☐	☐	☐	☐	☐	☐
…feeling part of your community?	c7n3	☐	☐	☐	☐	☐	☐	☐	☐	☐	☐	☐
…your life as a whole?	c7n4	☐	☐	☐	☐	☐	☐	☐	☐	☐	☐	☐
…the amount of spare time you have?	c7n5	☐	☐	☐	☐	☐	☐	☐	☐	☐	☐	☐

C8 Generally speaking, how much would you say that most people can be trusted?

c8 ☐1 Most people can be trusted ☐2 You must be wary of people at all times

C9 How much of an effect did the flood crisis in 2011 have on your physical possessions *(house or belongings)***?**

c9 ☐1 A lot ☐2 Some effect ☐3 Little effect ☐4 No effect

C10 How much of an effect did the flood crisis in 2011 have on your mental health?

c10 ☐1 A lot ☐2 Some effect ☐3 Little effect ☐4 No effect

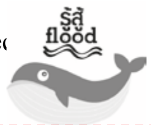

C11 In general in 2012 what impact have floods had on you when compared with 2011?

c11 ☐1 More than 2011 ☐2 Less than 2011 ☐3 About the same as 2011 ☐4 Did not have any effect

D Your food and physical activity

D1 How many serves of vegetables do you eat per day? d1 ☐☐ **serves/day** *eg if you eat 3 per day please put* 0 3

(for vegetables 1 serve = 1/2 cup of cooked vegetables or 1 cup of raw vegetables)

D2 How many serves of fruit do you eat per day? d2 ☐☐ **serves/day** *eg if you eat 5serves per day please put* 0 5

(for fruit 1 serve = 1 banana, 1 slice of papaya or 1 cup of diced pieces of fruit)

D3 How many teaspoons of fish sauce do you add to your food in an average day? d3 ☐☐ **teaspoons per day**

if you don't add fish sauce at all please put 0 0

D4 How many teaspoons of sugar do you add to your meals and drinks in an average day? d4 ☐☐ **teaspoons per day**

if you don't add sugar at all please put 0 0

D5 Have you ever seen "nutrition labels" on food?

d5 ☐1 I have seen them and have read them ☐2 I have seen them but have not yet read one ☐3 I am unaware of them

D6 How often do you use information from nutrition labels on food products to assist your food purchasing decisions?

d6 ☐1 Every time I shop ☐2 Often ☐3 Sometimes
☐4 Seldom ☐5 Never

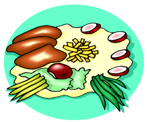

D7 How well do you understand the information presented on food "nutrition labels"?

d7
- 1 I understand fully
- 2 I understand most of the information
- 3 I understand some of the information
- 4 I do not understand the information but I know it has potential benefit
- 5 I don't understand the information or its potential benefit

D8 Would you like to see more nutrition labeling on foods?

d8
- 1 Yes
- 2 No
- 3 Don't know

D9 On average how often do you eat the following types of food? *(Please cross the one box which fits best for each food type)*

	Never or less than monthly	1-3 times/month	1-2 times/week	3-6 times/week	Daily or more
Food or dessert with coconut milk d9n1	1	2	3	4	5
Deep fried food d9n2	1	2	3	4	5
Instant foods eg instant noodles d9n3	1	2	3	4	5
Fermented/ salted raw food eg crab, fish d9n4	1	2	3	4	5
Fermented fruit/ vegetable d9n5	1	2	3	4	5
White rice or white sticky rice d9n6	1	2	3	4	5
Brown or combined brown and white rice d9n7	1	2	3	4	5
Fish and fish products d9n8	1	2	3	4	5
Soft drink (eg 7-Up, coke, pepsi) d9n9	1	2	3	4	5
Other sweetened drinks (eg iced tea or coffee, sweetened herb drinks) d9n10	1	2	3	4	5
Milk – fresh, carton or powder d9n11	1	2	3	4	5
Vitamins or food supplements d9n12	1	2	3	4	5
Fast food (Western style/farang) eg hamburger, pizza d9n13	1	2	3	4	5
Western bakery products eg cake, cookies d9n14	1	2	3	4	5

D10 On your past normal day (ie not a day off or weekend)**, how many times did you have a meal?** d10 [ ][ ] **times/day**

(please include meals and snacks)

D11 In the past 7 days, how many times did you eat a main meal alone? d11 [ ][ ] **times/day**

D12 When you eat alone do you eat more, less or the same as when you eat with others?

d12
- 1 More
- 2 Less
- 3 About the same

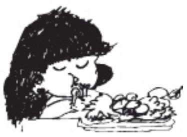

D13 How much of the following types of exercise do you do in a typical week?

(If you exercise 3 times per week please put 0 3 *If you don't do that type of exercise at all please put* 0 0 *)*

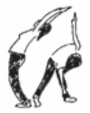

**Walking continuously <u>for at least 10 minutes</u>** *(for work, recreation, exercise or to get from place to place)*	d13n1	☐☐ times per week
**Vigorous physical activities for <u>more than 20 minutes</u>** *(that made you breathe harder or puff and pant)*	d13n2	☐☐ times per week
**Moderate physical activities for <u>more than 20 minutes</u>** *(like social tennis, golf, gentle swimming or work around the house or other work)*	d13n3	☐☐ times per week

D14 How often do you do household cleaning or gardening work?

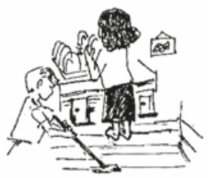

d14
- 1 Seldom or never
- 2 1-3 times/month
- 3 Once or twice/week
- 4 3-4 times/week
- 5 Everyday or almost everyday

D15 How many hours per day (ie per 24 hours) do you <u>usually</u> spend on the following activities?

Activities		Duration
**Standing for any purpose at all** *(eg for work, while socializing etc.)*	d15n1	☐☐ hours/day
**Sitting for any purpose** *(eg reading, resting, writing, thinking, TV, or computer)*	d15n2	☐☐ hours/day
**Sleeping** *(if you regularly sleep during the day include this also)*	d15n3	☐☐ hours/day
**Watching TV and/or playing computer games?**	d15n4	☐☐ hours/day

E Your injuries

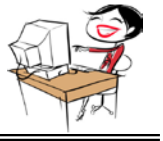

Your injuries – **traffic related**

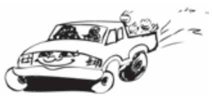

E1 In the <u>past 12 months</u>, how many times did you get injured in a traffic crash

e1
- 0 Never → Go to question **E7**
- 1 One
- 2 Two
- 3 Three
- 4 Four or more

E2 When you experienced your <u>most serious</u> traffic related injury did you receive medical care?

e2
- 1 Yes
- 2 No

E3 Did this injury <u>limit</u> your normal activities for one day or more?

e3
- 1 Yes
- 2 No

E4 When this injury occurred what was your role?

e4
- 1 Driver
- 2 Passenger
- 3 Pedestrian → Go to question **E6**

E5 Type of vehicle you were in or on as driver or passenger?

e5
- 1 Bicycle
- 2 Motorbike
- 3 Bus, van, tour coach
- 4 Car/pick-up
- 5 Other (eg train, plane, boat)

E6 What was the other party in the collision causing the traffic-related injury?

e6
- 1 Bicycle
- 2 Motorbike
- 3 Bus, van, tour coach
- 4 Car/pick-up
- 5 Other vehicle (eg train, boat)
- 6 Pedestrian
- 7 Animal (eg dog)
- 8 Other object not vehicle (eg tree, road, wall)

Your injuries – <u>non-traffic-related</u>

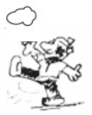

E7 In the <u>past 12 months</u>, how many times did you have a <u>NON-TRAFFIC</u> injury?

e7
- 0 Never → Go to question **F1**
- 1 One
- 2 Two
- 3 Three
- 4 Four or more

E8 When you experienced your <u>most serious</u> non-traffic-related injury did you receive medical care?

e8
- 1 Yes
- 2 No

E9 Did this injury limit your normal activities for one day or more?

e9
- 1 Yes
- 2 No

E10 <u>How</u> were you injured?

e10
- 1 Assault (punch, push or kick)
- 2 Other blunt (non-sharp) force
- 3 Stab/cut
- 4 Gunshot
- 5 Fall (not pushed)
- 6 Lifting heavy obje
- 7 Fire, heat, scald
- 8 Near-drowning
- 9 Bite or sting (animal, insect)
- 10 Poisoning
- 11 Choking
- 12 Other

E11 What was the location at which your most serious non-traffic related injury occurred?

e11
- 1 Home
- 2 Sports facility
- 3 Workplace (agricultural)
- 4 Workplace (non-agricultural)
- 5 Other

E12 What was <u>the nature</u> of your most serious non-traffic injury? *(You may choose more than one answer)*

- Fracture e12n1
- Sprain, strain or dislocation e12n2
- Cut, bite or open wound e12n3
- Bruise or superficial injury e12n4
- Burn/scald e12n5
- Concussion e12n6
- Organ system (internal) injury e12n7
- Other e12n8

E13 How did this non-traffic injury occur?

(Please choose only one answer)

- 1 Unintentional/ accident
- 2 Intentional by another person
- 3 Intentional (not involving another person)

e13

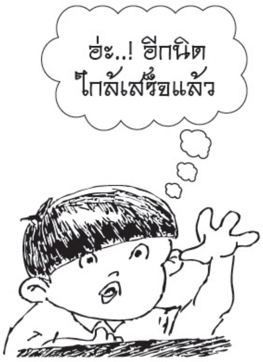

F Your Health History

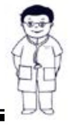

F1 Have you ever received a confirmed diagnosis from a doctor that you definitely have any of the following diseases?

Health condition		Definitely have disease	Doctor said I am at risk of the disease	Don't have the disease
Diabetes	f1n1	1	2	3
High cholesterol/high blood lipids	f1n2	1	2	3
High blood pressure	f1n3	1	2	3
Ischemic (coronary) heart disease	f1n4	1	2	3
Cerebrovascular disease (stroke)	f1n5	1	2	3
Liver cancer	f1n6	1	2	3
Lung cancer	f1n7	1	2	3
Stomach cancer	f1n8	1	2	3
Colon-rectum cancer	f1n9	1	2	3
Breast cancer	f1n10	1	2	3
Other cancers	f1n11	1	2	3
Kidney disease	f1n12	1	2	3
Other disease (specify).......f1n13x.....	f1n13	1	2	3

F2 What health insurance scheme/s covers you at present and for how long have you been covered? *(You may choose more than one answer)(If you have been covered by a scheme for less than one year please put* 0 1 *)*

Type of health insurance		Length of coverage	
☐ Don't have insurance	f2n1		
☐ Civil Servants Coverage	f2n2	f2n2y ☐☐	Yrs.
☐ Employer provided cover	f2n3	f2n3y ☐☐	Yrs.
☐ Private health insurance	f2n4	f2n4y ☐☐	Yrs.
☐ Social Security Scheme	f2n5	f2n5y ☐☐	Yrs.
☐ Universal Coverage Sche	f2n6	f2n6y ☐☐	Yrs.
☐ Other.......f2n7x........	f2n7	f2n7y ☐☐	Yrs.

F3 In the past 12 months how many times have you used the following types of health services? *(You may choose more than one answer)*

Health service type	Number of visits (past 12 months) eg if you visited once in the past year please put 0 1 times	
Government health centre	f3n1 ☐☐	times
Community hospital	f3n2 ☐☐	times
Private health clinic	f3n3 ☐☐	times
Government hospital	f3n4 ☐☐	times
Private hospital	f3n5 ☐☐	times
Traditional medicine	f3n6 ☐☐	times
Pharmacy	f3n7 ☐☐	times
Other.......f2n8x........	f3n8 ☐☐	times

G Smoking, alcohol and transport

G1 Are you a current smoker?

g1 [1] No [2] Yes and I smoke [|] cigarettes per day g1n

G2 Please describe your current alcohol drinking?

g2
- [1] Don't drink
- [2] Used to drink but quit
- [3] Drink in social situations, about [|] glasses/week g2n3
- [4] Current regular drinker of about [|] glasses/ day g2n4

G3 In the past 12 months have you ever driven a vehicle after consuming 3 or more glasses of alcohol?

g3
- [1] Yes
- [2] No
- [3] Don't normally drive

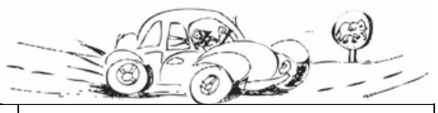

G4 In the past 12 months, for personal transport how often did you…?

		Always	Sometimes	Never	Not applicable	
Use car safety belt (front seat)	g4n1	1	2	3	4	No safety belt or don't ride in front seat
Use car safety belt (back seat)	g4n2	1	2	3	4	No safety belt or don't ride in back seat
Ride on back step of "song thaew"	g4n3	1	2	3	4	Don't use "song thaew"
Ride in back of open truck/pick up	g4n4	1	2	3	4	Don't use such vehicle
Use motorcycle helmet	g4n5	1	2	3	4	Don't use motorcycle
Ride on motorcycle with 3 or more peo	g4n6	1	2	3	4	Don't use motorcycle

H Birth and contraception *(for cohort member women only to answer)*

(Male cohort members please ignore this section)

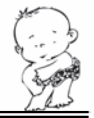

H1 How many babies have you ever given birth to? [|] h1 Babies *(If you have never given birth please put* [0|0] *)*

H2 Please tell us about your past and present use of the following contraceptives (If you have used for less than one year please put [0|0])

Have you ever taken or used the following types of hormonal contraceptives?		Age started using?	Age past used? (*or age now if still using*)	How long did you use it altogether? (*don't count periods of non-use*)
**Oral contraceptive pill** h2n1	[1] No<br>[2] Yes →	h2n1a yrs	h2n1b yrs	h2n1c yrs
**Injections every three months (depo provera)** h2n2	[1] No<br>[2] Yes →	h2n2a yrs	h2n2b yrs	h2n2c yrs
**Contraceptive implant under the skin (may past 3-5 years)** h2n3	[1] No<br>[2] Yes →	h2n3a yrs	h2n3b yrs	h2n3c yrs

Thank you very much for your kind cooperation 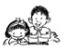

Supplement: S2 File — (PDF) [file pone.0189574.s002.pdf]
